# Giant Vesicles Encapsulating Aqueous Two-Phase Systems: From Phase Diagrams to Membrane Shape Transformations

**DOI:** 10.3389/fchem.2019.00213

**Published:** 2019-04-09

**Authors:** Yonggang Liu, Reinhard Lipowsky, Rumiana Dimova

**Affiliations:** ^1^State Key Laboratory of Polymer Physics and Chemistry, Changchun Institute of Applied Chemistry, Chinese Academy of Sciences, Changchun, China; ^2^Department of Theory and Bio-Systems, Max Planck Institute of Colloids and Interfaces, Potsdam, Germany

**Keywords:** phase diagram, membrane shape transformation, giant vesicles, aqueous two-phase systems, dextran, poly(ethylene glycol), wetting, membrane tubes

## Abstract

In this review, we summarize recent studies on giant unilamellar vesicles enclosing aqueous polymer solutions of dextran and poly(ethylene glycol) (PEG), highlighting recent results from our groups. Phase separation occurs for these polymer solutions with concentration above a critical value at room temperature. We introduce approaches used for constructing the phase diagram of such aqueous two-phase system by titration, density and gel permeation chromatography measurements of the coexisting phases. The ultralow interfacial tension of the resulting water-water interface is investigated over a broad concentration range close to the critical point. The scaling exponent of the interfacial tension further away from the critical point agrees well with mean field theory, but close to this point, the behavior disagrees with the Ising value of 1.26. The latter discrepancy arises from the molar mass fractionation of dextran between coexisting phases. Upon encapsulation of the PEG–dextran system into giant vesicles followed by osmotic deflation, the vesicle membrane becomes completely or partially wetted by the aqueous phases, which is controlled by the phase behavior of the polymer mixture and the lipid composition. Deflation leads to a reduction of the vesicle volume and generates excess area of the membrane, which can induce interesting transformations of the vesicle morphology such as vesicle budding. More dramatically, the spontaneous formation of many membrane nanotubes protruding into the interior vesicle compartment reveals a substantial asymmetry and spontaneous curvature of the membrane segments in contact with the PEG-rich phase, arising from the asymmetric adsorption of polymer molecules onto the two leaflets of the bilayers. These membrane nanotubes explore the whole PEG-rich phase for the completely wetted membrane but adhere to the liquid-liquid interface as the membrane becomes partially wetted. Quantitative estimates of the spontaneous curvature are obtained by analyzing different aspects of the tubulated vesicles, which reflect the interplay between aqueous phase separation and spontaneous curvature. The underlying mechanism for the curvature generation is provided by the weak adsorption of PEG onto the lipid bilayers, with a small binding affinity of about 1.6 k_B_T per PEG chain. Our study builds a bridge between nanoscopic membrane shapes and membrane-polymer interactions.

## Introduction

Phase separation can occur when solutions of two different polymers or a polymer and a salt are mixed above a certain concentration in water. These aqueous two-phase systems (ATPSs) provide a particularly mild environment with extremely low interfacial tension on the order of 1–100 μN/m, which enable many applications of ATPS in biotechnology and bioengineering (Walter et al., 1985; Albertsson, 1986). One such system of great interest is provided by mixing aqueous solutions of dextran and polyethylene glycol (PEG). These solutions undergo phase separation above the critical concentration at a certain temperature, yielding two coexisting phases in equilibrium with each phase containing predominantly one of the polymer species and water. Phase separation in polymer solutions depends on the thermodynamic properties of the system, which is theoretically described by the Flory-Huggins theory (Flory, 1941, 1953; Huggins, 1941). When the entropy of mixing is not sufficient to compensate the enthalpy of demixing, the polymer solutions undergo phase separation.

Recently, renewed interest in PEG–dextran systems arose because of its potential biotechnological applications, as well as its suitability as a model system for mimicking the crowded environment in cells (Dimova and Lipowsky, 2012, 2017; Keating, 2012). The PEG–dextran ATPS was encapsulated into giant unilamellar vesicles (GUVs), cell-sized containers (Dimova et al., 2006; Walde et al., 2010; Dimova, 2012, 2019). The group of Keating initiated the study of aqueous phase separation in GUVs (Helfrich et al., 2002), and observed the asymmetric protein microcompartmentation in these systems which resembles the crowded environment of the cytosol (Long et al., 2005). The partitioning of biomolecules in ATPS is influenced by the affinities of these molecules being separated to the coexisting phases or the liquid-liquid interface, as well as by the physico-chemical properties of the employed ATPS itself (Zaslavski, 1995), which requires a detailed and quantitative characterization of its phase behavior. During the last decade, these hybrid soft matter systems containing both membranes and polymers are investigated experimentally (Li et al., 2008, 2011, 2012; Long et al., 2008; Kusumaatmaja et al., 2009; Andes-Koback and Keating, 2011; Liu et al., 2016) and theoretically (Lipowsky, 2013, 2014, 2018). A number of interesting phenomena, such as vesicle budding (Long et al., 2008; Li et al., 2012), wetting transitions (Li et al., 2008; Kusumaatmaja et al., 2009), division of vesicles (Andes-Koback and Keating, 2011), and formation of membrane nanotubes (Li et al., 2011; Liu et al., 2016), have been observed. All these phenomena were governed by the interplay between polymer-membrane interactions and the fluid-elastic properties of the membrane (Lipowsky, 2013, 2014, 2018). Precise experimental studies of the aqueous phase separation and the resulting aqueous phases are challenging, but are required to fully understand their role in the associated membrane transformations. This review focuses on precisely this topic, highlighting results from our groups that have been obtained over the past decade.

The text is organized as follows. We first discuss the phase diagram of the PEG–dextran system. More specifically, we introduce a density method for the measurement of the tie lines between the coexisting phases, and compare it with a method based on gel permeation chromatography (GPC). We then compare the scaling exponent of the interfacial tension to the values obtained in mean field theory and in the Ising model, and correlate the discrepancy of the Ising value in the vicinity of the critical point to the molar mass fractionation of dextran between coexisting phases. Afterwards, we focus on membrane-associated effects (such as wetting and morphological changes) in GUVs encapsulating ATPS. The observed complete-to-partial wetting transition of the giant vesicle membrane by the PEG-rich phase is discussed by introducing a hidden material parameter, the intrinsic contact angle (Kusumaatmaja et al., 2009), which characterizes the affinity of the phases to the membranes but has not been directly measured by optical microscopy. We then discuss the formation of membrane nanotubes resulting from the deflation of giant vesicles encapsulating aqueous mixture of dextran and PEG. Theoretical analysis of the GUV shapes with nanotubes protruding into the interior of the vesicles revealed the presence of a negative spontaneous curvature (Li et al., 2011). Depending on the properties of the aqueous phases and the vesicle membranes, three different tube patterns have been observed within vesicles of three distinct morphologies (Liu et al., 2016). Quantitative estimation of the spontaneous curvature is obtained by image analysis of the vesicle shapes, for membranes of different lipid compositions with distinct fluid-elastic properties. The molecular mechanism underlying the observed curvature generation is provided by the weak adsorption of PEG molecules onto the membranes, according to theoretical considerations, control experiments with PEG solution, and molecular dynamics simulations. Finally, we discuss possible future directions in the field.

## Phase Diagram of the PEG–Dextran Systems

**Figure 1:**
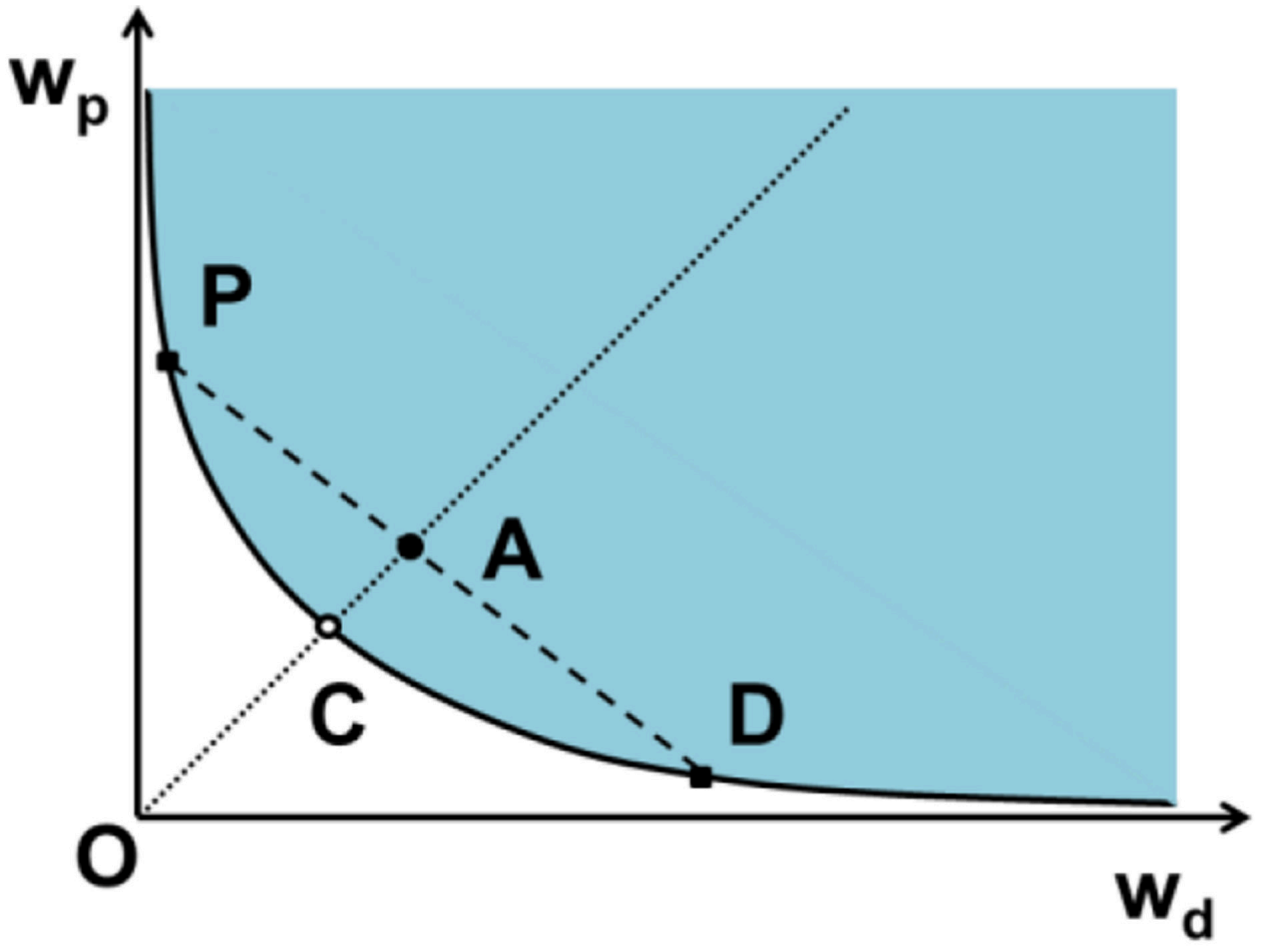
Schematic phase diagram for an aqueous two-phase system, in our case dextran (d) and PEG (p), by plotting the weight fraction of PEG *w*_p_ as a function of the weight fraction of dextran *w*_d_. The phase diagram is divided into regions of two-phase coexistence (blue) and one homogeneous phase (white) by the binodal (solid curve). The critical point C, at which the volumes of the two coexisting phases become identical, is located infinitely close to and above the binodal. The mixture A with the same polymer ratio as the critical point, located above the binodal undergoes phase separation and forms two coexisting phases with compositions D and P in equilibrium, which are dextran-rich and PEG-rich phases, respectively. Solutions with composition lying on this tie line (dashed line) separate into coexisting phases with the same final compositions (D and P) but different volume fractions. The composition difference of the coexisting phases is characterized by the length of the tie line DP, which becomes shorter at lower polymer concentration and converges to a single point called the critical demixing point (C).

At a certain temperature, the phase diagram for an aqueous solution of dextran and PEG depends on their weight fractions *w*_d_ and *w*_p_. The diagram includes the binodal (the boundary of the two-phase coexistence region), the critical point and the tie lines, as illustrated in Figure 1. The phase diagram is divided by the binodal curve into a region of polymer concentrations that will form two immiscible aqueous phases (above the binodal in Figure 1) and one homogeneous phase (at and below the binodal in Figure 1). A tie line connects two points of the binodal, which represent the final compositions of the polymer components in the coexisting phases. Also located on the binodal is the critical demixing point. Above this point but close to the binodal (see Figure 1), the compositions and volumes of both phases are nearly identical. Different methods have been proposed to construct the phase diagram of PEG–dextran system (Hatti-Kaul, 2000). Below, we will review some of them.

### Binodal and Critical Point

**Figure 2:**
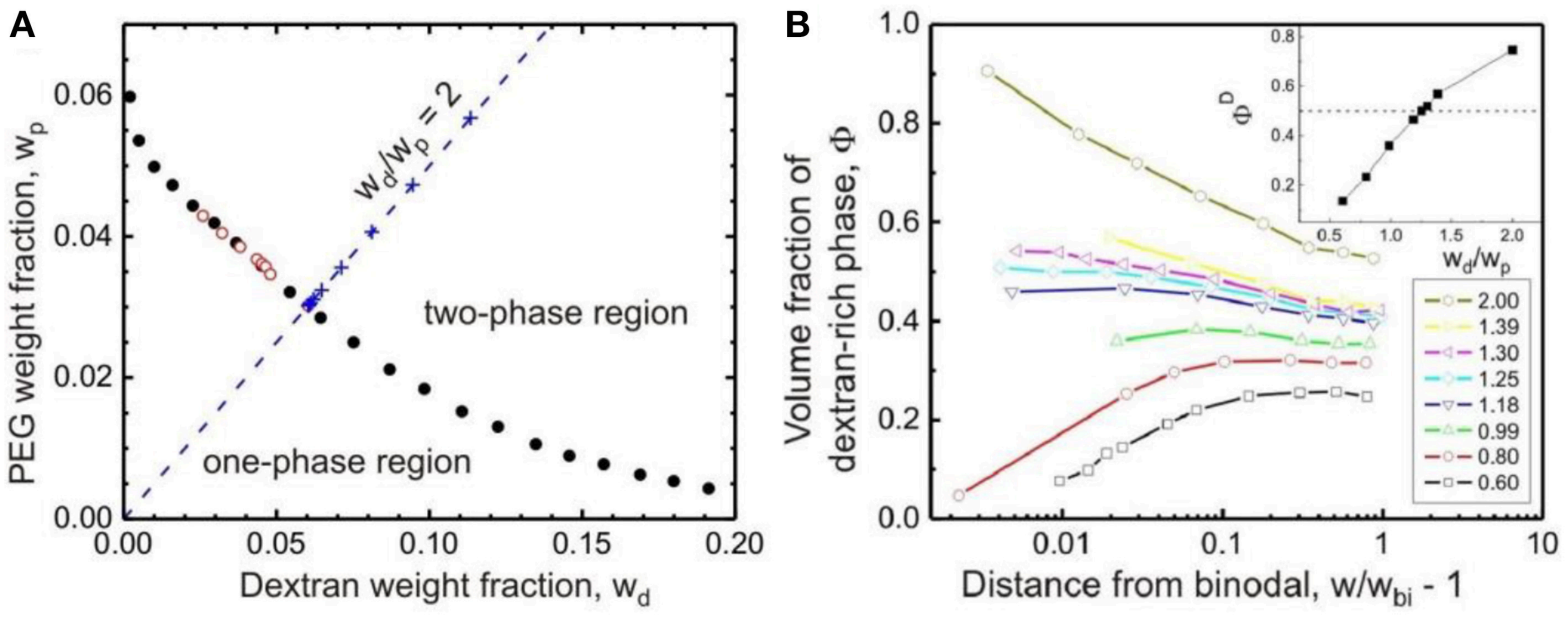
**(A)** Binodal of the aqueous solution of dextran (molar mass between 400 and 500 kg/mol) and PEG (molar mass 8 kg/mol) at 24 ± 0.5°C obtained by titration from the one-phase to the two-phase region (solid circles) and vice versa (open circles). The “+” symbols are experimental points along the titration trajectory with *w*_d_/*w*_p_ = 2.0 (dashed line). The intersection of such a trajectory with the binodal defines the polymer weight fraction *w*_bi_. **(B)** Volume fraction Φ^D^ of the dextran-rich phase as a function of the normalized distance from the binodal, *w*/*w*_bi_ – 1, for polymer solutions of different weight ratios *w*_d_/*w*_p_ between dextran and PEG ranging from 0.60 to 2.00. See the lower inset with the color code. The upper inset shows the dependence of the volume fraction Φ^D^ on the weight ratio *w*_d_/*w*_p_ very close to the phase boundary at *w*/*w*_bi_ = 1.02. For Φ^D^ = 0.50 (dashed line), the polymer weight ratio *w*_d_/*w*_p_ = 1.25 was found. Reprinted with permission from Liu et al. (2012). Copyright (2012) American Chemical Society.

The binodal determined by cloud-point titration is shown in Figure 2A for aqueous solutions of dextran (with weight-average molar mass *M*_w_ = 400–500 kg/mol) and PEG (with *M*_w_ = 8 kg/mol) (Liu et al., 2012). The aqueous mixture of dextran and PEG undergoes phase separation when the total polymer weight fractions exceed a few percent. Titration experiments from the one-phase to the two-phase region, or the other way around, lead to the same phase boundary.

The critical point of the system, at which the volumes of the coexisting phases are equal, can be estimated by gradually approaching the binodal via titration of the PEG–dextran mixture in the two-phase region with water. In this experiment, a series of mixtures of dextran and PEG solutions are prepared at certain weight ratios *w*_d_/*w*_p_, and the volume fractions of the coexisting phases are measured by bringing the system stepwise to the binodal. Using data obtained from titration trajectories with different values of *w*_d_/*w*_p_, one can find the weight ratio *w*_d_/*w*_p_ at which the two phases have equal volumes in the vicinity of the binodal, in this case *w*_d_/*w*_p_ = 1.25 is found, as shown in Figure 2B (Liu et al., 2012). Carefully studying solutions with such a weight ratio close to the binodal provides an estimate of the polymer composition of the critical point, which is located at a total polymer weight fraction *w*_cr_ = 0.0812 ± 0.0002. The critical concentration for phase separation of the studied PEG–dextran system is then given by *c*_cr_ = ρ_cr_*w*_cr_ = 0.0829 ± 0.0002 g/mL with ρ_cr_ being the solution mass density at the critical point.

It should be mentioned that there is a temperature dependence for the phase diagram of the PEG–dextran system (Helfrich et al., 2002), one can therefore use either temperature or concentration as experimental control parameters for the phase state of the PEG–dextran system. Additionally, new phase diagrams should be measured when new lots of polymer are used, due to the batch-to-batch differences of the polymers in molar mass distributions, even if they are obtained from the same manufacturer (Helfrich et al., 2002).

### Tie Line Determination

To assess the polymer concentrations and build the tie lines in ATPSs, one has to separate the phases and measure some physical properties that related to the polymer concentrations. For the PEG–dextran system, one normally measures the optical activity and the refractive index of the solutions, because dextran is optically active but PEG is not. Then dextran concentrations in the coexisting phases are obtained from the known specific rotation of dextran, while the PEG concentrations are determined after subtracting the contribution of dextran to the solution refractive index. To make it simpler, a gravimetric method had been employed for the tie line determination of ATPS containing a PEG polymer and a salt, by forcing the end points of the tie-line on a binodal determined separately (Merchuk et al., 1998). However, for ATPS containing polymers with large dispersities, the tie line end points deviates from the binodal, and the mismatch grows with increasing polymer dispersity. It makes the gravimetric method not applicable to the PEG–dextran systems, because the generally available dextran has a broad molar mass distribution. Below we show that the tie lines of an ATPS can be accurately determined by density and gel permeation chromatography measurements of the coexisting phases.

#### Density Method

This method for determining the tie lines of ATPS is based on accurate density measurements of the coexisting phases (Liu et al., 2012). Here we assume that the specific volume of the aqueous polymer solution is the sum of the contributions from all components. Then, the mass density ρ of the mixture is related to the specific volume of each component via

(1) $$\begin{array}{r} \frac{1}{\rho }=\left(1-w_{d}-w_{p}\right)\nu_{s}+w_{d}\nu_{d}+w_{p}\nu_{p} \end{array}$$

**Figure 3:**
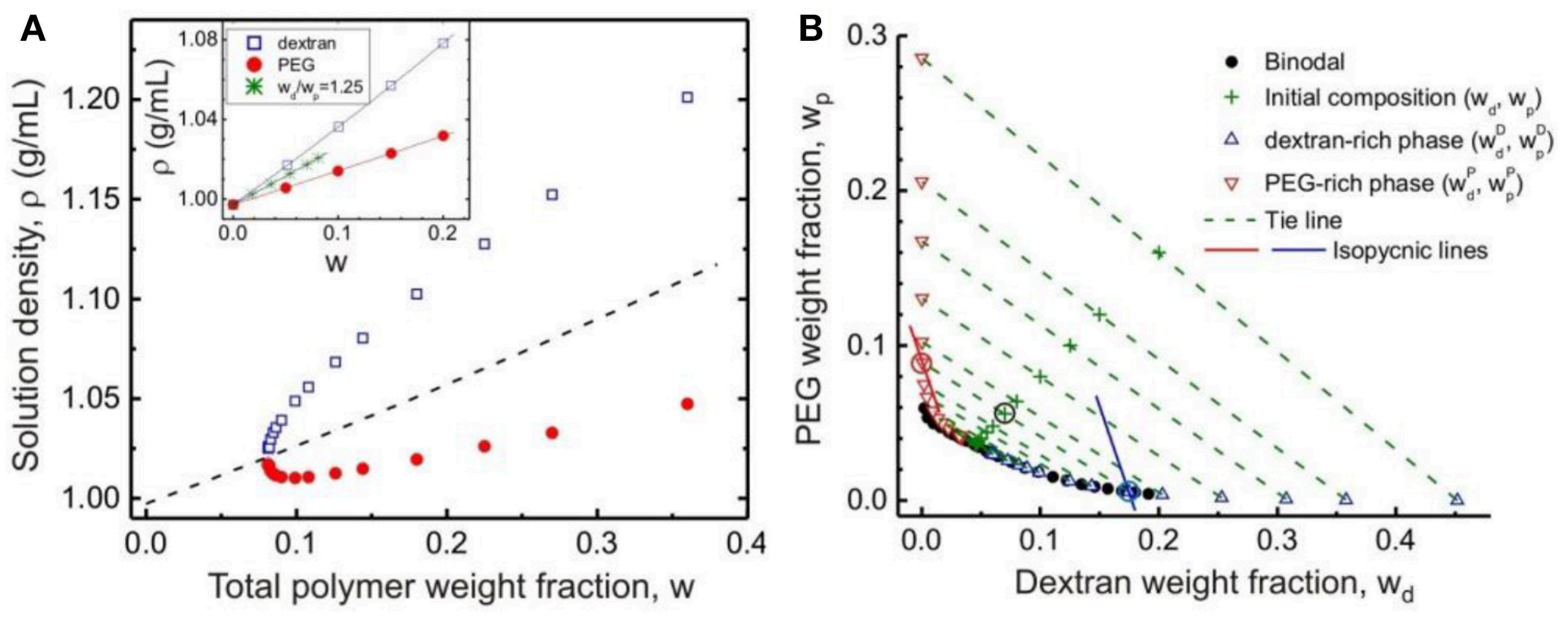
**(A)** Densities of the coexisting dextran-rich (open squares) and PEG-rich (solid circles) phases for polymer solutions with weight ratio *w*_d_/*w*_p_ = 1.25 as functions of the total initial polymer weight fraction w. The dashed line is the calculated density of the polymer solution with *w*_d_/*w*_p_ = 1.25. In the inset, the densities of pure dextran and pure PEG solutions and their mixtures with *w*_d_/*w*_p_ = 1.25 in the one-phase region are plotted as functions of the total polymer weight fraction *w*. The lines are fits to Equation (1) with specific volumes *v*_d_ = 0.62586 ± 0.00046 mL/g and *v*_p_ = 0.83494 ± 0.00043 mL/g. **(B)** Tie lines in the PEG–dextran phase diagram at 24 ± 0.5°C. The solid circles show the data for the experimentally measured binodal. The compositions of the initial solutions (with weight ratio *w*_d_/*w*_p_ = 1.25) for which the phase densities after phase separation were measured are indicated by “+” symbols. The end points of the respective tie lines consist of upward-pointing triangles indicating the compositions of the dextran-rich phases and downward-pointing triangles indicating the compositions of the PEG-rich phases. The solid lines represent two examples of isopycnic lines calculated following Equations (2) and (3) for the initial solution composition indicated with an encircled “+” symbol in the graph: (*w*_d_, *w*_p_) = (0.0700, 0.0560). The intersections of the isopycnic lines with the binodal yield the compositions of the two phases, also encircled. Reprinted with permission from Liu et al. (2012). Copyright (2012) American Chemical Society.

Here the specific volume of water, dextran and PEG at 24°C are found to be *v*_s_ = 1.00271 mL/g, *v*_d_ = 0.62586 mL/g and *v*_p_ = 0.83494 mL/g, respectively (see inset of Figure 3A).

In Liu et al. (2012), we prepared PEG–dextran solutions in the concentration range *w*_cr_ < *w* < 0.36 at the same weight ratio *w*_d_/*w*_p_ as for the critical point. These solutions were kept at a constant temperature of 24°C for a few days to reach equilibrium before the coexisting phases separated and their densities accurately measured by a density meter. As expected, the top PEG-rich phase always has a lower density than the bottom dextran-rich phase, and the density difference between the coexisting phases vanish at the critical point (Figure 3A). The normalized distance of the corresponding tie line from the critical point is taken to be the reduced concentration $\varepsilon \equiv \frac{c}{c_{cr}}-1$, which lies in the range of 0 < ε < 3.82.

The compositions of the dextran-rich (D) and PEG-rich (P) phases, are then determined based on their densities ρ^D^ and ρ^P^, respectively. By rewriting Equation (1), the PEG weight fractions of the coexisting phases, *w*${}_{\text{p}}^{\text{D}}$ and *w*${}_{\text{p}}^{\text{P}}$, are related to the corresponding dextran weight fractions, $w_{\text{d}}^{\text{D}}$ and $w_{\text{d}}^{\text{P}}$, via (Liu et al., 2012):

(2) $$\begin{array}{r} w_{p}^{D}=\frac{1}{\nu_{p}-\nu_{s}}\left[\frac{1}{\rho^{D}}-\nu_{s}-\left(\nu_{d}-\nu_{s}\right)w_{d}^{D}\right] \end{array}$$

for the dextran-rich phase, and

(3) $$\begin{array}{r} w_{p}^{P}=\frac{1}{\nu_{p}-\nu_{s}}\left[\frac{1}{\rho^{P}}-\nu_{s}-\left(\nu_{d}-\nu_{s}\right)w_{d}^{P}\right] \end{array}$$

for the PEG-rich phase, respectively.

Equations (2) and (3) represent straight isopycnic lines in the *w*_d_-*w*_p_ plane with a constant slope of −(ν_*d*_ − ν_*s*_)/(ν_*p*_ − ν_*s*_), and the intercepts of these lines reflect the different values of the phase densities ρ^D^ and ρ^P^. The compositions of the coexisting dextran-rich and PEG-rich phase can be then estimated from the intersections of these isopycnic lines with the binodal established in section Binodal and Critical Point.

The accuracy of this density-based method in constructing the tie lines is demonstrated by the close proximity of the coordinates (*w*_d_, *w*_p_) for the starting mixtures to the corresponding tie lines, as shown in Figure 3B. The tie lines determined by the density method are in excellent agreement with reported tie lines obtained with traditional methods for similar PEG–dextran systems at comparable temperatures. Below we will show that the density method can be further validated by an independent method based on quantitative GPC measurements of the coexisting phases.

#### GPC Method

The density method is relatively simple to determine the tie lines of ATPS. However, it relies on the assumption that the tie line end points coincide with the predetermined binodal, which is a good approximation for ATPS with polymers of narrow dispersities. For PEG–dextran systems with two polymer species which can be completely separated by GPC, the compositions of the coexisting phases can be directly quantified by GPC with a single concentration detector (Connemann et al., 1991; Zhao et al., 2016a,b). Below we give the details of this method.

**Figure 4:**
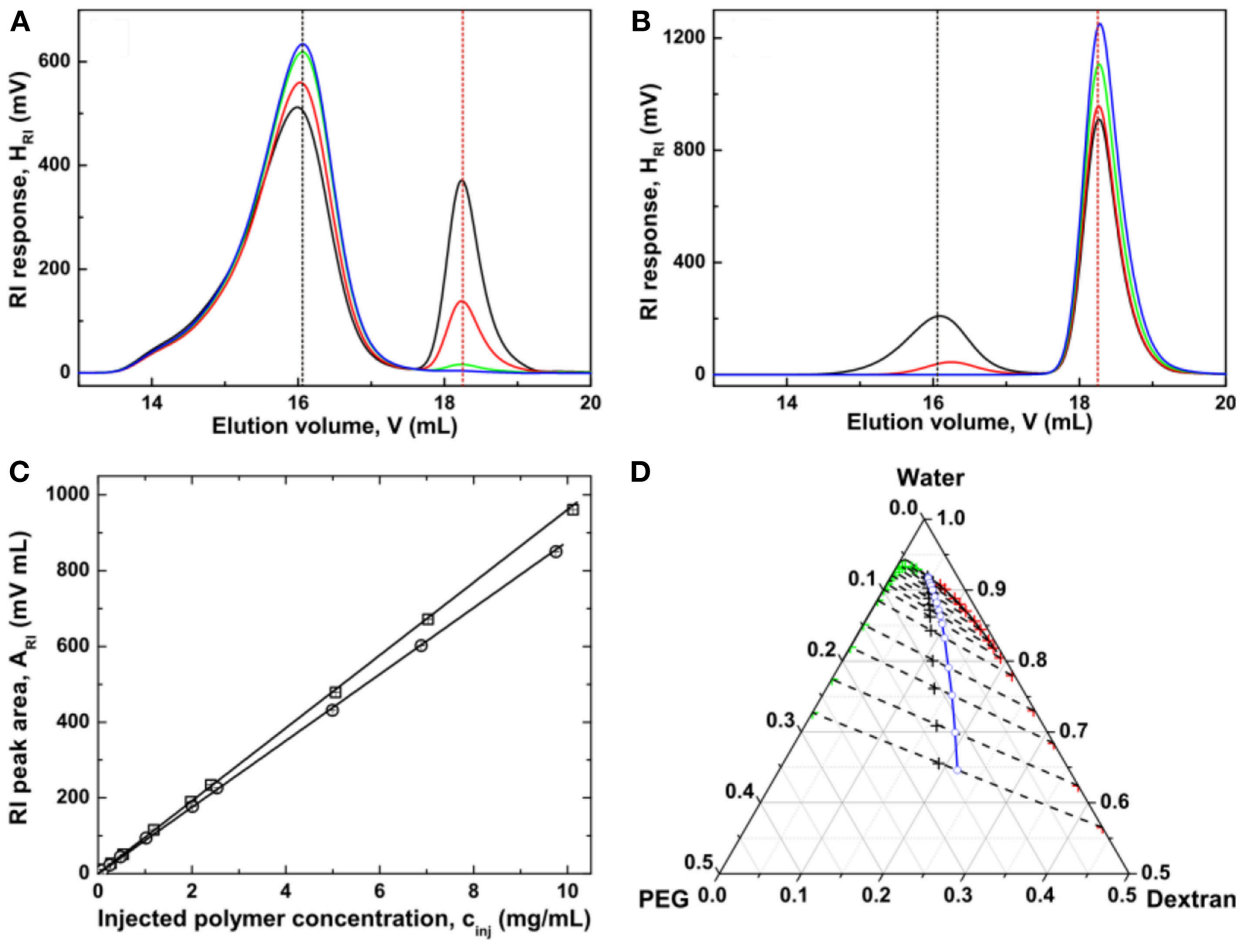
GPC chromatograms of coexisting dextran-rich **(A)** and PEG-rich phases **(B)** at ε = 0.030 (black), 0.200 (red), 0.982 (green), and 2.087 (blue). The peak retention volumes of the native dextran and PEG are 16.06 mL (black dashed line) and 18.25 mL (red dashed line), respectively. **(C)** Dependence of the RI peak area *A*_RI_ on polymer concentration *c*_inj_ of the solutions injected into the size-exclusion chromatography columns for dextran (squares) and PEG (circles). **(D)** The resulting phase diagram of the PEG–dextran–water system. In the phase diagram, the cloud point curve is shown as a solid curve. The compositions of the initial solutions for which size-exclusion chromatography measurements after phase separation were performed are indicated by black crosses. The end points of the respective tie lines (dashed lines) consist of red crosses indicating the compositions of the dextran-rich phases and green crosses indicating the compositions of the PEG-rich phases. The midpoints (blue circles) of the tie lines were extrapolated to the binodal to determine the critical point. Adapted with permission from Zhao et al. (2016b). Copyright (2016) Chem. J. Chinese Universities.

To quantify polymer concentrations within the two coexisting phases of an ATPS, the polymer solutions are typically diluted and their GPC chromatograms are recorded, with baseline separation of dextran from PEG on a differential refractive index (RI) detector (Zhao et al., 2016b). It can be seen from Figures 4A,B that further away from the critical point, more dextran molecules are accumulating in the dextran-rich phase, while more PEG molecules are partitioning into the PEG-rich phase. At sufficient distance from the critical point, no dextran molecules are present in the PEG-rich phase, and PEG molecules are completely absent in the dextran-rich phase. The polymer compositions in the coexisting phases can be directly obtained from their peak areas, with the pre-established concentration dependences of the RI peak areas for dextran and PEG, respectively (Figure 4C). It is found that the tie line end points superpose to the binodal curve, with an exception of those data for the PEG-rich phases in the vicinity of the critical point (Figure 4D). This discrepancy is most probably due to molar mass fractionation of dextran between the coexisting phases (see section Molar Mass Fractionation).

Interestingly, the tie lines established by the density and GPC methods agreed well with each other. The density method requires the density measurements of the phases together with a pre-established binodal, which makes it a simple and convenient method. The GPC method requires the chromatography measurements of all phases and accurate calibration of the RI detector for both components. Although the GPC method is tedious and time-consuming, it does not depend on the binodal curve. More importantly, molar mass distribution and molar mass averages of each polymer species in the coexisting phases can be obtained by the GPC method (see section Molar Mass Fractionation). It should be noted, however, that the GPC method with RI as the concentration detector is only applicable to certain ATPSs, whose components can be separated into two peaks by the GPC columns without polymer adsorption. The coupling to a laser light scattering detector gives additional information on molar mass of the components. If the elution peaks of these two polymer components are overlapping, one must use two concentration detectors, for example a RI and an additional optical rotation detector, to quantify the compositions of the polymer mixtures (Edelman et al., 2003a,b).

### Molar Mass Fractionation

Since dextran and PEG components in the coexisting phases are completely separated from each other by the GPC columns (Figures 4A,B), one can also determine the molar mass distributions of each component after calibrating the system either with narrow polymer standards or by a laser light scattering detector (Zhao et al., 2016a). We first take a look at the dextran component in the coexisting phases. Inspection of the GPC chromatograms (Figures 4A,B) indicates that the relative intensity of the elution peak of dextran in the PEG-rich phase is much less than that in its coexisting dextran-rich phase. Additionally, the elution peak of dextran in the PEG-rich phase is shifting toward higher retention volume, indicating a lower molar mass than that in the dextran-rich phase. Further away from the critical point, the difference for the dextran elution peaks between the two coexisting phases increases. The evolution of the PEG elution peaks shows a different behavior. Although the relative intensity of PEG elution peak in the dextran-rich phase is lower than that in the PEG-rich phase, there is however, hardly any change in retention volumes for PEG components in the two coexisting phases. It indicates that PEG components in the two coexisting phases have similar molar mass, albeit less PEG is distributed into the dextran-rich phase.

**Figure 5:**
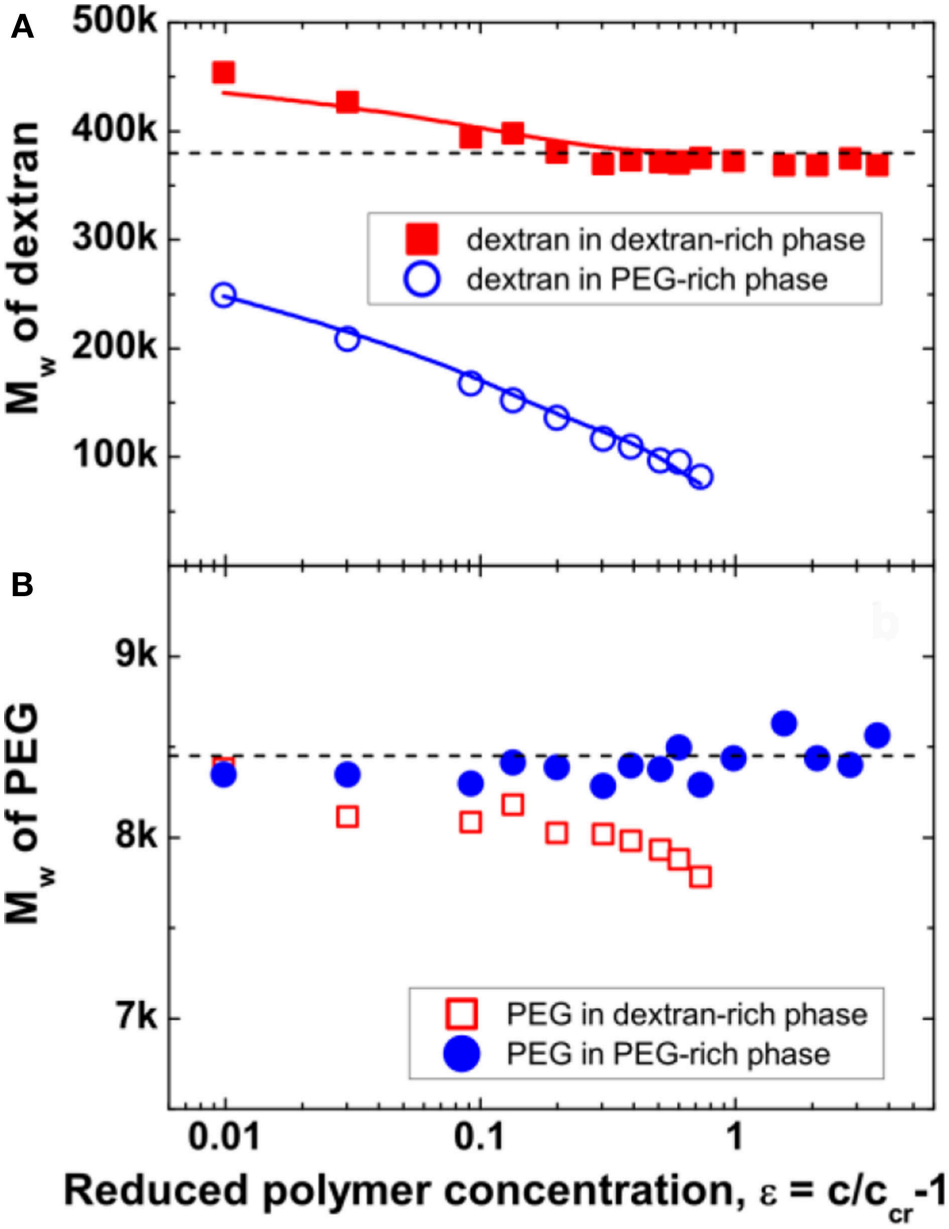
Weight-average molar mass *M*_w_ of dextran **(A)** and PEG **(B)** in the dextran-rich (squares) and PEG-rich (circles) phases. The dashed lines indicate the average molar mass *M*_w_ = 380 kg/mol for dextran and *M*_w_ = 8.45 kg/mol for PEG, respectively. The values of *M*_w_ for dextran in the two phases, which were obtained from the calculated molar mass distribution of dextran, are shown as solid lines in **(A)**. Reprinted from Zhao et al. (2016a), Copyright (2016), with permission from Elsevier.

Quantitative calculation of the molar mass and polymer dispersities for dextran and PEG in the coexisting phases are obtained from GPC measurements and the results are shown in Figure 5. For the system explored here, it is found that the weight-average molar mass *M*_w_ of dextran in the dextran-rich phase is significantly larger than that in its coexisting PEG-rich phase (Figure 5A). As the polymer concentration increases, the *M*_w_ of dextran in the dextran-rich phase approaches the value of the original dextran with *M*_w_ = 380 kg/mol, while the *M*_w_ of dextran in the PEG-rich phase shows a continuous decrease down to 82.2 kg/mol at ε = 0.73. This is because the used dextran had a broad molar mass distribution, characterized by the dispersity index *M*_w_*/M*_n_, the ratio between the weight-average molar mass *M*_w_ and the number-average molar mass *M*_n_, of 2.19. With more dextran of high molar mass component partitioning into the dextran-rich phase, the molar mass of dextran component in the PEG-rich phase decreases. It also leads to an *M*_w_ value larger than that of the native dextran in the dextran-rich phase, agreeing with the tiny shift of the dextran peak to lower retention volume (Figure 4A). However, when the initial polymer concentration is sufficiently high, no dextran is found in the PEG-rich phase, leading to the same *M*_w_ value of dextran in the dextran-rich phase as for native dextran. In contrast, PEG in the two coexisting phases have similar weight-average molar masses, close to the value of the original PEG with *M*_w_ = 8.45 kg/mol (Figure 5B), and such behavior is independent of the initial polymer concentration. This is due to the narrow dispersity of the employed PEG, with *M*_w_*/M*_n_ = 1.11. In ATPS with broad molar mass distributions for both polymers, molar mass fractionation of both components is observed (Edelman et al., 2003a,b).

With the compositions of the coexisting phases accurately measured, we can obtain the distribution coefficient *f*_x_(N) of each polymer component between the coexisting phases. According to the Flory-Huggins theory, the distribution coefficient, also called the degree of fractionation, is defined as (Flory, 1953):

(4) $$\begin{array}{r} f_{x}\left(N\right)\equiv \frac{c_{x,poor}\left(N\right)}{c_{x,rich}\left(N\right)}=\exp\left(-\sigma_{x}N\right) \end{array}$$

where *c*_x,poor_(*N*) and *c*_x,rich_(*N*) are the concentrations of component *x* (in our case, dextran or PEG) containing *N* monomers in the phases poor and rich in *x* component, respectively. Theory predicted an exponential decay behavior for the degree of fractionation *f*_x_(*N*) as a function of chain length *N* (Flory, 1953; Koningsveld et al., 2001), suggesting that the longer the *x*-chain, the less it distributed in the *x*-poor phase. The separation parameter σ_x_ represents the free energy change per monomer during transferring a chain of length *N* between the liquid-liquid phases.

**Figure 6:**
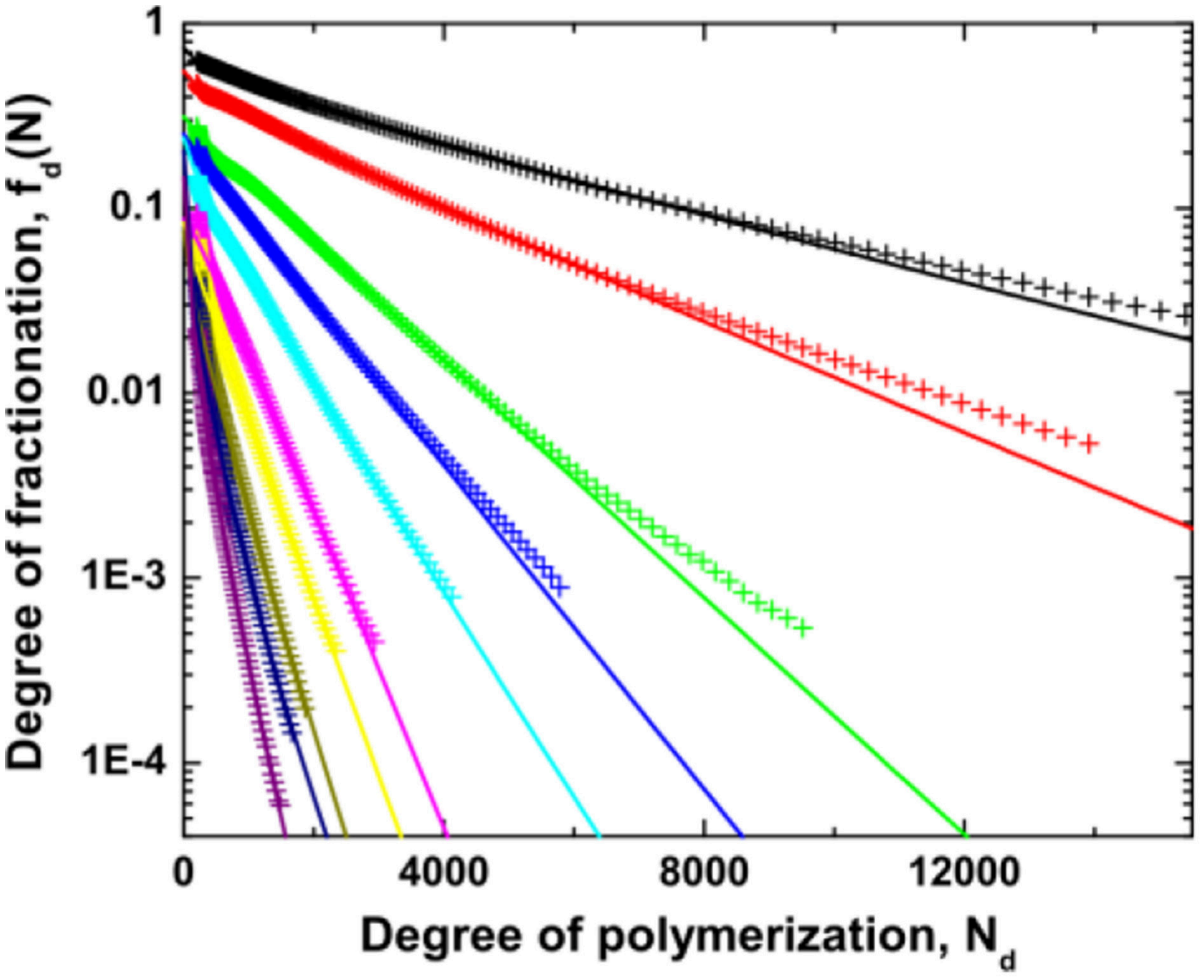
Degree of fractionation *f*_d_(*N*) for dextran as a function of the degree of polymerization *N*_d_ of dextran for different values of the reduced polymer concentration $\varepsilon \equiv \frac{c}{c_{cr}}-1$ which is varied from 0.01 (top right) to 0.73 (bottom left). The lines are fits to the data by the empirical relation $f_{d}\left(N\right)=A\times exp\left(-\sigma_{d}N-\sigma_{d2}N^{0.5}\right)$. Reprinted from Zhao et al. (2016a), Copyright (2016), with permission from Elsevier.

In Figure 6, the degree of fractionation *f*_d_(*N*) for dextran is plotted vs. the chain length *N*_d_ at different distance ε from the critical point. An exponential dependence is observed over a certain range of chain length for all values of ε, although the data deviate slightly. However, there are two distinct differences of the experimental results with the mean field prediction. First, the degree of fractionation starts to deviate from the exponential dependence at a certain chain length. Second, the value extrapolated to *N*_d_ = 0 does not reach the expected value of 1, implying that the mean field theory is insufficient to quantitatively describe the degree of fractionation for dextran in ATPS. Such a discrepancy has been observed in previous experiments (Edelman et al., 2003a,b), as well as in computer simulations (van Heukelum et al., 2003). The data can be fitted to an empirical relation by introducing additional fitting parameters *A* and σ_d2_, as shown in Figure 6.

For the current ATPS containing dextran chains with a large dispersity and near-monodisperse PEG chains, molar mass fractionation leads to a chain length dependent redistribution of dextran chains between the coexisting phases. High molar mass dextran chains are enriched in the dextran-rich phase and depleted from the PEG-rich phase, leading to a significant decrease of the *M*_w_ of dextran in the PEG-rich phase and a slight increase of that in the coexisting dextran-rich phase. This is the underlying origin for the mismatch between the binodal curve and the end points of the tie lines for the PEG-rich phase in the vicinity of the critical point. As a result, the compositions of the PEG-rich phase locate above the binodal (Figure 3B). It is also expected that the compositions of the corresponding dextran-rich phases lie slightly below the binodal, which is not observable in experiments. The results are in good agreement with previous studies about polymer dispersity effect on the tie lines (Kang and Sandler, 1988). Therefore, to obtain the tie line for such an ATPS system by the density method close to the critical point (Liu et al., 2012), the composition for the dextran-rich phase can be determined from the intersection of the corresponding isopycnic line with the binodal. However, the composition for the PEG-rich phase must be estimated from the intersection of its isopycnic line with a straight line passing through the composition of the initial polymer solution and that of the dextran-rich phase.

## Interfacial Tension and Scaling Laws

The interfacial tension between coexisting phases of ATPS is on the order of 1–100 μN/m, which can be determined by measuring the equilibrium shape of liquid-liquid interface under some external forces, such as gravity or centrifugal force, as well as by methods based on time evolution of the interface shape (Tromp, 2016). Different techniques, such as the drop volume method (Mishima et al., 1998), drop retraction analysis (Ding et al., 2002), sessile and pendant drop shape analysis (Atefi et al., 2014), capillary length analysis (Vis et al., 2015) and spinning drop method (Ryden and Albertsson, 1971; Liu et al., 2012), have been employed for the interfacial tension measurement of the water-water interfaces.

**Figure 7:**
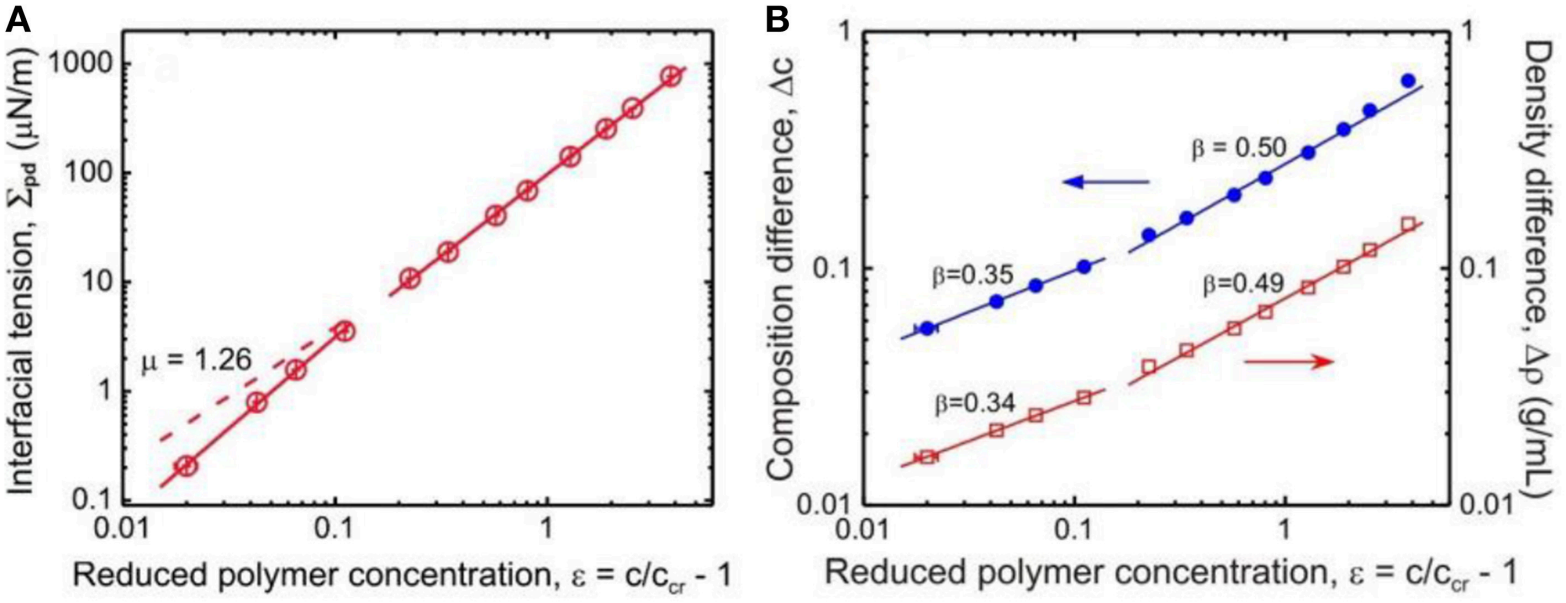
**(A)** Interfacial tension Σ_pd_ between coexisting dextran-rich and PEG-rich phases as functions of the reduced polymer concentration ε = *c*/*c*_cr_ – 1. The solid lines are fits to the data with exponent of 1.67 ± 0.10 for 0.02 < ε < 0.12, and 1.50 ± 0.01 for 0.2 < ε < 3. The dashed line shows the expected asymptotic behavior with μ = 1.26. **(B)** Composition difference Δ*c* (solid circles) and density difference Δρ (open squares) of the coexisting phases as functions of the reduced polymer concentration ε. In the concentration range 0.02 < ε < 0.12, the fits to the data give for the scaling exponent β a value of 0.337 ± 0.018 as estimated from the density difference dependence or 0.351 ± 0.018 as estimated from the composition difference dependence, while in the range 0.2 < ε < 3 we obtain β = 0.491 ± 0.014 from the density difference dependence or β = 0.503 ± 0.018 from the composition difference dependence. Reprinted with permission from Liu et al. (2012). Copyright (2012) American Chemical Society.

Here, we summarize some data for the interfacial tension Σ_pd_ between the coexisting liquid-liquid phases of the PEG–dextran system obtained by a spinning drop tensiometer (Liu et al., 2012) (see Figure 7A). In this broad concentration range, the interfacial tension increases by 4 orders of magnitude with increasing distance from the critical point, i.e. from 0.21 μN/m at reduced polymer concentration ε = 0.02, to 769 μN/m at ε = 3.82.

Quantitative description of the phase separation of polymer solution in the vicinity of the critical point remains as an interesting problem in polymer physics. The phase separation of polymer solution is usually studied at temperature *T* close to its critical demixing temperature *T*_c_. Various physical quantities, including the susceptibility χ, the correlation length ξ, the order parameters, and the interfacial tension Σ, have been studied in details for different polymer solutions (Sanchez, 1989; Widom, 1993). Several theoretical models, such as the mean field theory, the Ising model, and the crossover theory, have been developed to explain the observed scaling behaviors of the these properties, depending on proximity to the critical point as characterized by the reduced temperature τ = |1 – *T*/*T*_c_|. A good example is provided by accurate light scattering measurements of polymer solutions close to *T*_c_ (Melnichenko et al., 1997; Anisimov et al., 2002), where the scaling for the susceptibility χ ~ τ^−γ^ with γ changed from 1.24 to 1, and that for the correlation length ξ ~ τ^−ν^ with ν decreased from 0.63 to 1/2, respectively. The crossover from the Ising model to the mean field behavior occurs at a correlation length scale on the order of the chain size of the polymers. Measurement of the coexistence curves for polymer solutions also revealed such a crossover for the composition difference Δφ ~ τ^β^ with the exponent β changed from 0.326 to 1/2 (Dobashi et al., 1980). The crossover for the interfacial tension Σ ~ τ^μ^ in exponent μ is theoretically predicted but its experimental verification is still lacking. Instead, scaling exponents μ ranged from 1.17 to 1.60 have been reported in the literature for a few polymer systems close to *T*_c_ (Shinozaki et al., 1982; Heinrich and Wolf, 1992; Widom, 1993), where the crossover from the Ising value 1.26 to the mean field value 3/2 were not observed.

One can use a similar approach for ATPS of dextran and PEG, by scaling analysis of the interfacial tension and the order parameters vs. the reduced concentration ε (Liu et al., 2012). The scaling exponent μ of the interfacial tension $\Sigma_{\text{pd}}\sim \varepsilon^{\mu }$ shows a crossover behavior depending on proximity to the critical point (Figure 7A). Further away from the critical point, the obtained value μ = 1.50 ± 0.01 is in excellent agreement with mean field theory. However, closer to this point, the increased value of 1.67 ± 0.10 deviates significantly from the Ising value 1.26. In contrast, as the critical point is approached, the scaling exponent β of the order parameters, does exhibit the expected crossover from mean field value of β = 1/2 to Ising value 0.326 (see Figure 7B).

**Figure 8:**
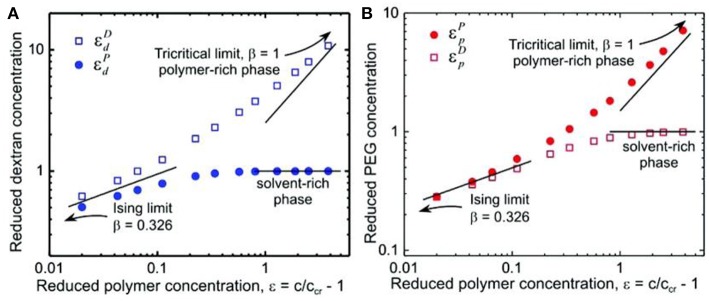
Logarithmic scaling plots of phase-coexistence curves for **(A)** dextran and **(B)** PEG. The solid lines are guides to the eye with scaling exponents of 0.326 for the Ising model limit and 1 for the tricritical limit and a constant of 1 for the solvent-rich phase. Reprinted with permission from Liu et al. (2012). Copyright (2012) American Chemical Society.

Such a crossover from mean field to Ising behavior is further supported by the normalized coexistence curve of the studied PEG–dextran system, which has a similar shape with the prediction from the crossover theory based on near-tricritical-point (theta point) Landau expansion renormalized by fluctuations (Anisimov et al., 2000). As shown in Figure 8, the reduced polymer concentration approximates the Ising limit with scaling exponent β = 0.326 in the vicinity of the critical point, where the correlation length of the concentration fluctuations is larger than the polymer molecular size (Liu et al., 2012). However, the data at the highest polymer concentration approaches the tricritical mean field limit with β = 1, indicating that the polymer molecular size is larger than the correlation length. Therefore, the crossover theory from critical Ising behavior to tricriticality of polymer solutions can also be applied to the PEG–dextran system in our study.

The discrepancy for the scaling exponent of the interfacial tension close to the critical point might arise from the molar mass fractionation of dextran. As shown in Figure 5A, in the vicinity of the critical point, the *M*_w_ of dextran in the dextran-rich phase is larger than that of the original dextran, leading to a reduction of the interfacial tension. While further away from the critical point, the *M*_w_ of dextran in the dextran-rich phase is similar to that of the original dextran and there is no influence on the interfacial tension. Therefore, the scaling exponent μ is unaffected in the mean field region, but an increased value of 1.67 is observed in the Ising limit region.

## Wetting of Membranes by ATPS

**Figure 9:**
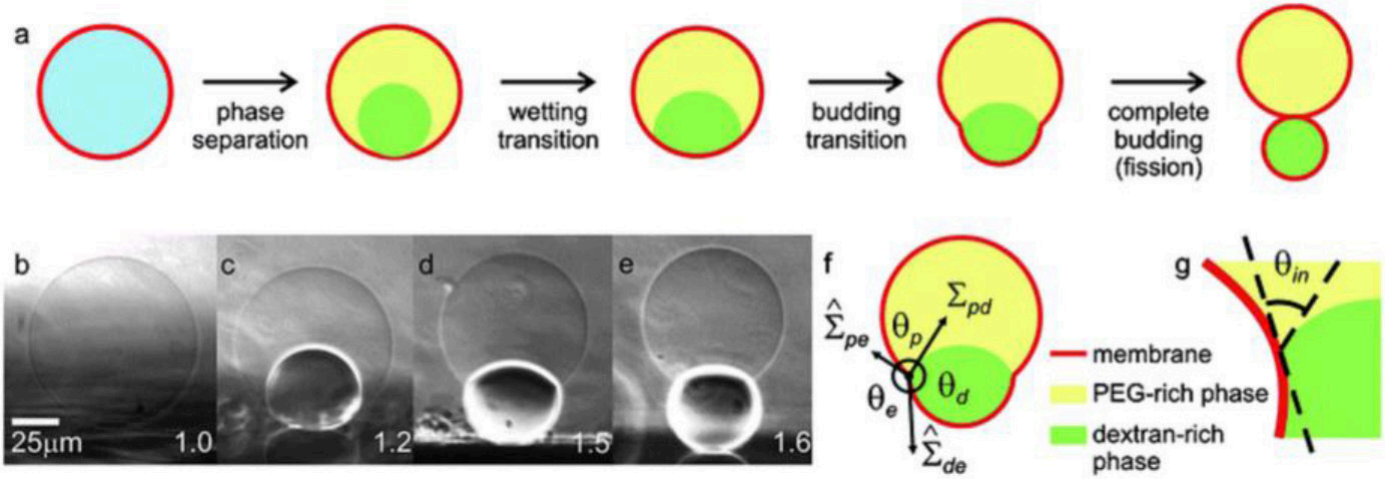
Response of giant vesicles encapsulating ATPS when exposed to osmotic deflation. **(a)** Schematic illustration of the steps upon deflation: phase separation within the vesicle, wetting transition, vesicle budding, and fission of the enclosed phases into two membrane-wrapped droplets. **(b–e)** Side-view phase contrast images of a vesicle sitting on a glass substrate. The vesicle contains the PEG–dextran ATPS. After phase separation **(b,c)**, the interior solution consists of two liquid droplets consisting of PEG-rich and dextran-rich phases, respectively. Further deflation of the vesicle causes the dextran-rich droplet to bud out as shown in **(d,e)**. The numbers on the snapshots indicate the osmolarity ratio between the external medium and the initial internal polymer solution. In the sketch in **(f)**, the three effective contact angles as observed with optical microscopy are indicated, as well as the two membrane tensions and the interfacial tension Σ_pd_. The contact line is indicated by the circled dot. The intrinsic contact angle θ_in_, which characterizes the wetting properties of the membrane by the PEG-rich phase at the nanometre scale, is sketched in **(g)**. Reproduced from Dimova and Lipowsky (2012) with permission from the Royal Society of Chemistry.

The phase separation process and its consequences on membranes in contact with the two phases can be directly observed when the aqueous polymer solutions of dextran and PEG in the one-phase region are encapsulated within GUVs (Li et al., 2011; Liu et al., 2016). In these studies, the polymer solutions undergo phase separation when the system is brought into two-phase region via osmotic deflation by exposing the vesicles to a hypertonic medium, i.e., vesicles deflation (note that the lipid membrane is permeable to water, but not to the polymers, which become concentrated as water permeates out). The deflation not only leads to a reduction of the vesicle volume and the formation of two immiscible aqueous phases within vesicles, but also generates excess area of the membrane. This results in a variety of interesting changes in the vesicle morphology, such as vesicle budding (Long et al., 2008; Li et al., 2012), wetting transition (Li et al., 2008; Kusumaatmaja et al., 2009), and complete budding of the vesicles (Andes-Koback and Keating, 2011) as schematically shown in Figure 9a. The overall vesicle shape can be observed from confocal microscopy cross sections of the vesicles, as well as from side-view phase-contrast images (Figures 9b–e) on a horizontally aligned microscope (Li et al., 2011; Liu et al., 2016).

Depending on the phase state of encapsulated ATPS and the interactions between the aqueous phases with the vesicle membrane, the liquid droplet may exhibit zero or non-zero contact angles corresponding, respectively, to complete or partial wetting. In the vicinity of the critical point, the membrane is completely wetted by the PEG-rich phase, while further away from this point, both phases partially wet the membranes. For GUV encapsulating PEG–dextran mixture, a complete-to-partial wetting transition had been observed for a number of lipid compositions (Li et al., 2008, 2011; Kusumaatmaja et al., 2009; Liu et al., 2016).

When the vesicle membrane becomes partially wetted by the aqueous phases, it is separated into two different segments: one is in contact with the PEG-rich phase, and the other with the dextran-rich phase. These two membrane segments and the pd interface, i.e., the interface between the PEG-rich and dextran-rich phases, have spherical cap morphology. Then from the geometry of the vesicle, three effective contact angles θ_d_, θ_p_, and θ_e_ can be obtained, with θ_p_+θ_d_+θ_e_ = 2π, as shown in Figure 9f. The force balance of the tensions (of the two membrane segments and the pd interface) at the three-phase contact line implies that the three tensions form a triangle, which leads to the relations (Kusumaatmaja et al., 2009; Li et al., 2011; Lipowsky, 2018):

(5) $$\begin{array}{r} {\hat{\Sigma }}_{\text{pe}}=\Sigma_{\text{pd}}\sin\theta_{\text{d}}/sin\theta_{\text{e}} \end{array}$$

(6) $$\begin{array}{r} {\hat{\Sigma }}_{\text{de}}=\Sigma_{\text{pd}}\sin\theta_{\text{p}}/sin\theta_{\text{e}} \end{array}$$

between these tensions and the effective contact angles. Here, ${\hat{\Sigma }}_{\text{pe}}$ is the tension of the pe membrane segment in contact with the PEG-rich phase, and ${\hat{\Sigma }}_{\text{de}}$ is the tension of the de membrane segment in contact with the dextran-rich phase; e describes the external phase outside the vesicle. The membrane tensions can then be calculated from the interfacial tension Σ_pd_ as measured in section Interfacial Tension and Scaling Laws and the effective contact angles, which are obtained from the microscopy images.

When viewed with optical resolution, the shape of budded vesicles as in Figures 9d,e exhibits a kink at the three-phase contact line. However, the bending energy of the membrane kink would become infinite if it persists to smaller length scales (Kusumaatmaja et al., 2009). Therefore, the membrane in vicinity of the contact line should be smoothly curved when viewed with a super resolution microscopy (Zhao et al., 2018), which reveals the existence of an intrinsic contact angle θ_in_, as shown schematically in Figure 9g (Kusumaatmaja et al., 2009). If the two membrane segments have identical curvature-elastic properties, the force balance along the three phase contact line gives (Lipowsky, 2018)

(7) $$\begin{array}{r} {\hat{\Sigma }}_{\text{de}}-{\hat{\Sigma }}_{\text{pe}}=W_{\text{de}}-W_{\text{pe}}=\Sigma_{\text{pd}}\text{cos}\theta_{\text{in}} \end{array}$$

Here *W*_de_ and *W*_pe_ are adhesive strengths of the two membrane segments. Therefore, the intrinsic contact angle θ_in_ is related to three material parameters, the adhesive strengths *W*_de_ and *W*_pe_ and the interfacial tension of the liquid-liquid interface (Lipowsky, 2018). However, if the two membrane segments have the same elastic properties but different spontaneous curvatures, additional terms resulting from the different curvature-elastic properties emerges, and the truncated force balance relation as given by Equation (7) may lead to unreliable estimates for the intrinsic contact angle (Lipowsky, 2018).

## Formation of Membrane Nanotubes in Vesicle Encapsulating ATPS

Osmotic deflation of giant vesicles enclosing PEG–dextran solutions, can lead to spectacular shape changes as evidenced by the formation of many membrane nanotubes protruding into the interior of the vesicle. Because the membrane is not pulled by an external force, the driving force for the spontaneous tubulation of vesicles is provided by a membrane tension generated from a substantial spontaneous curvature, which is much larger than the curvature of the GUV membranes. The spontaneous curvature is the preferred curvature the membrane would adopt when left at rest. It can be modulated by various factors such as leaflet compositional asymmetry, adsorption and depletion of molecular species and ions (Lipowsky, 2013; Bassereau et al., 2018). In the system considered here, the spontaneous curvature arising from the polymer-membrane interactions can be estimated using three different and independent methods of image analysis on the vesicle morphologies (Liu et al., 2016). A combination of experiment, theoretical analysis and computer simulation reveals the molecular mechanism for the membrane spontaneous curvature generated in the system of giant vesicles encapsulating ATPS.

### Three Patterns of Flexible Nanotubes

**Figure 10:**
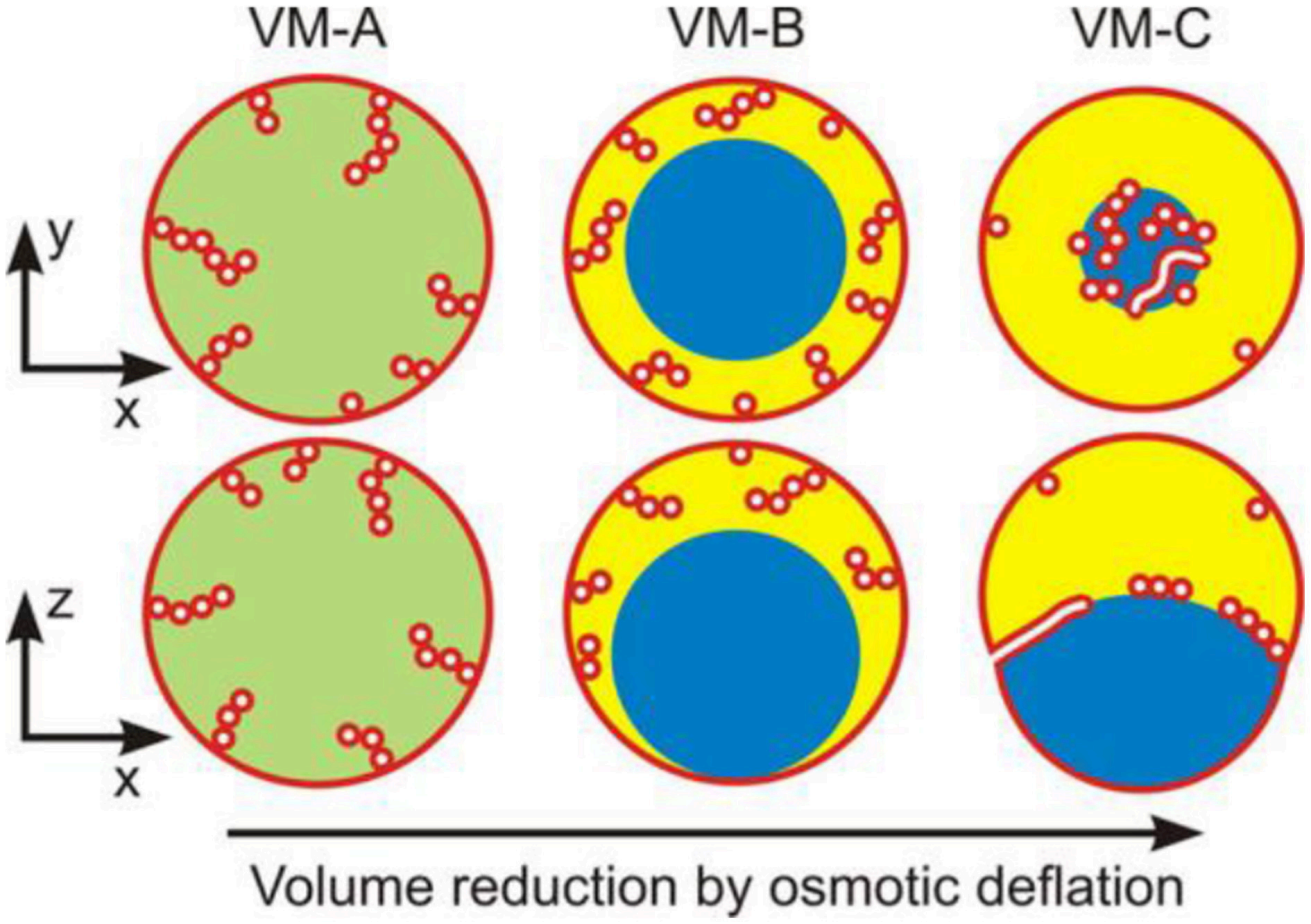
Three nanotube patterns (VM-A, VM-B, and VM-C) corresponding to the distinct vesicle morphologies (VM) observed along the deflation path: Schematic views of horizontal *xy*-scans (top row) and of vertical *xz*-scans (bottom row) across the deflated vesicles. In all cases, the tubes are filled with external medium (white); the membrane is shown in red. For the VM-A morphology, the interior polymer solution is uniform (green), whereas it is phase-separated (blue and yellow) for the morphologies VM-B and VM-C, with complete and partial wetting, respectively, of the membrane by the PEG-rich aqueous phase (yellow). For the VM-B morphology, the nanotubes explore the whole PEG-rich (yellow) droplet but stay away from the dextran-rich one (blue). For the VM-C morphology, the nanotubes adhere to the interface between the two aqueous droplets forming a thin and crowded layer over this interface. It is expected that in the VM-A and VM-B morphologies, these nanotubes are necklace-like consisting of a number of small spheres connected by narrow or closed membrane necks, while in the VM-C morphology, cylindrical tubes with a uniform diameter along the nanotubes co-exist with the necklace-like ones. Reprinted with permission from Liu et al. (2016). Copyright (2016) American Chemical Society.

**Figure 11:**
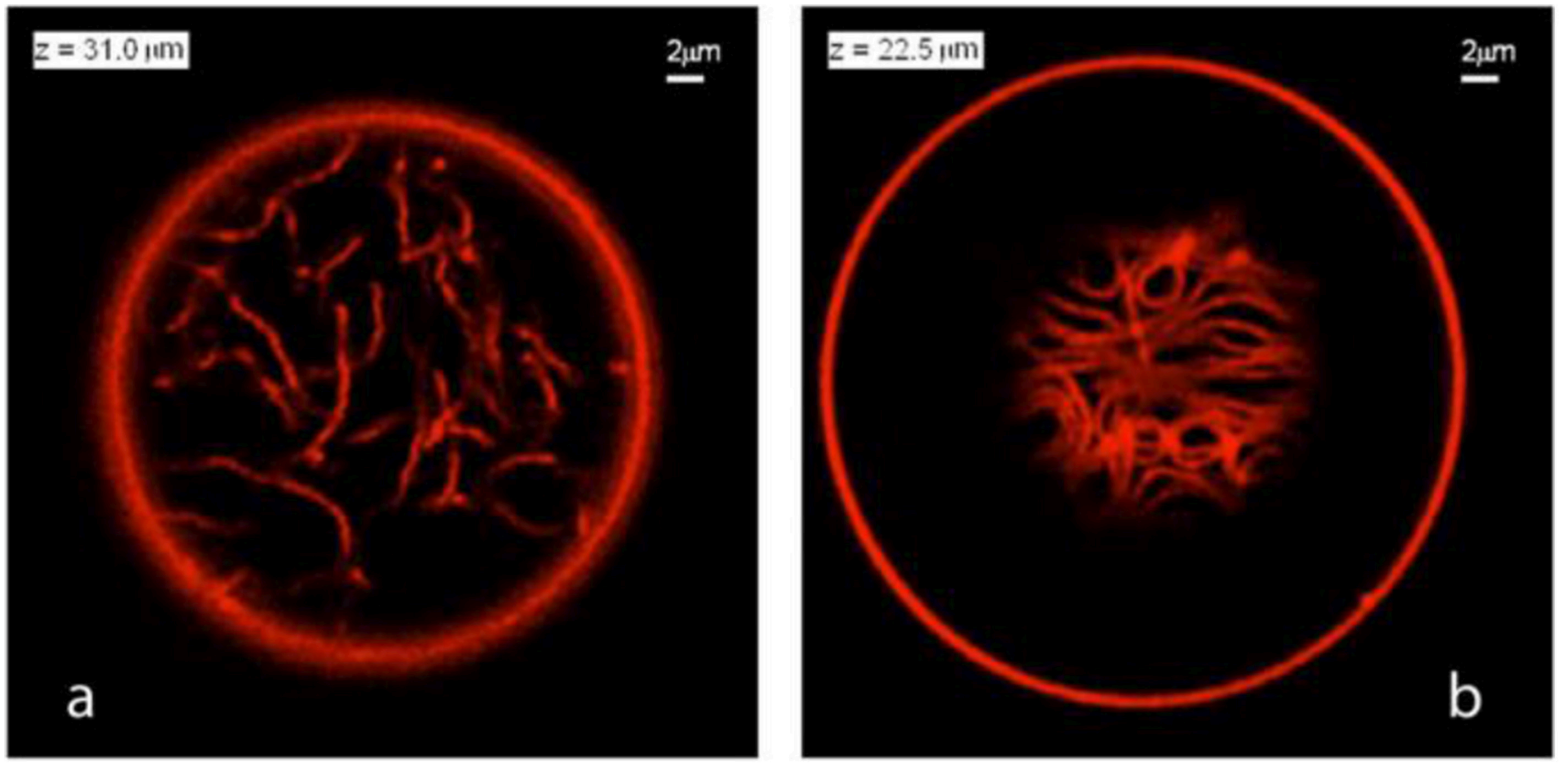
Nanotube patterns within Ld-phase vesicles as observed for the VM-B and VM-C morphologies corresponding to complete and partial wetting of the membranes. **(a)** Disordered pattern corresponding to a confocal *xy*-scan of the VM-B morphology. Because the Ld membrane is completely wetted by the PEG-rich phase, the nanotubes explore the whole PEG-rich droplet but stay away from the dextran-rich phase located below the imaging plane. **(b)** A layer of densely packed tubes as visible in an *xy*-scan of the VM-C morphology. As a result of partial wetting, the nanotubes now adhere to the pd interface between the two aqueous droplets and form a thin layer in which crowding leads to short-range orientational order of the tubes. Note that the tube layer is only partially visible because the pd interface is curved into a spherical cap. Both in **(a,b)**, the diameter of the tubes is below the diffraction limit, but the tubes are theoretically predicted to have necklace-like and cylindrical shapes in panels **(a,b)**, respectively. Reprinted with permission from Liu et al. (2016). Copyright (2016) American Chemical Society.

**Figure 12:**
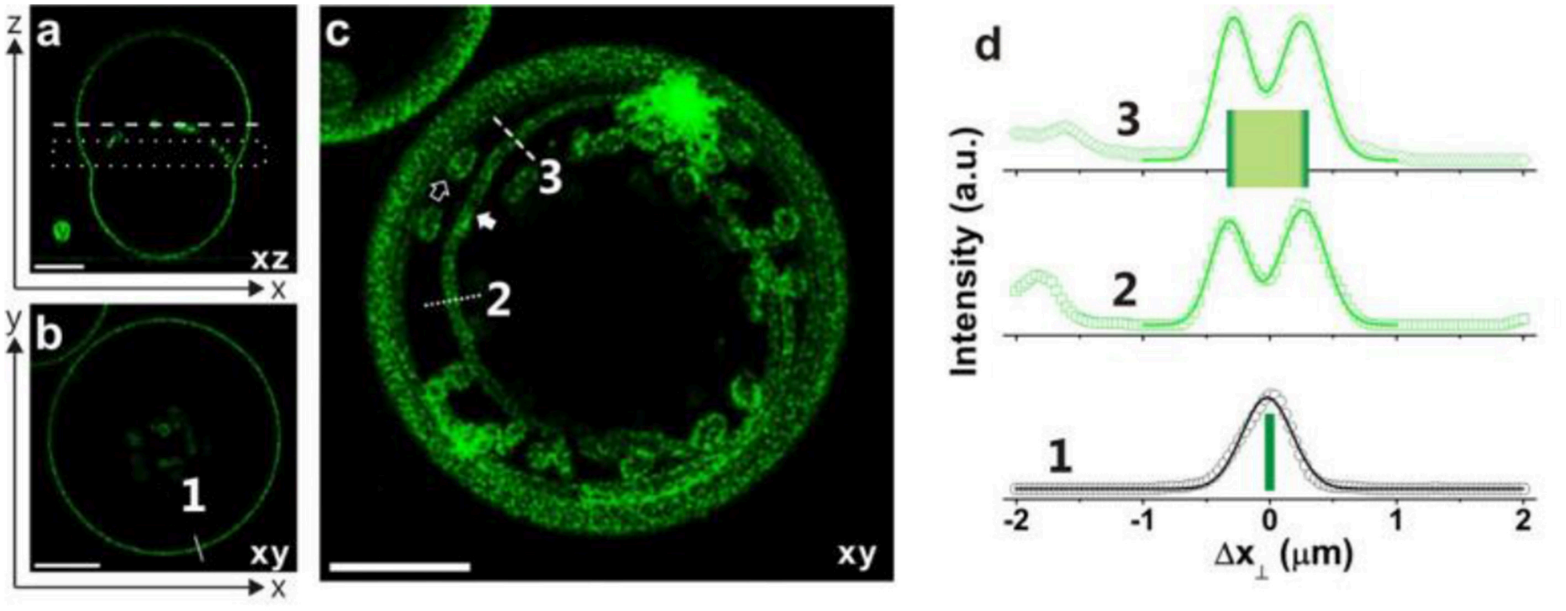
Necklace-cylinder tube coexistence for giant vesicles with Lo membranes: **(a)** confocal *xz*-scan; **(b)** confocal *xy*-scan corresponding to the dashed line in panel a; **(c)** superposition of 6 confocal *xy*-scans located in the dotted rectangle in panel a. This projection image reveals the coexistence of several long cylindrical tubes and several short necklace-like tubes. All scale bars are 10 μm. **(d)** Fluorescent intensity along the solid white line 1 in panel **(b)** perpendicular to the GUV contour and along the dotted and dashed white lines 2 and 3 in panel **(c)** across a cylindrical tube. The quantity Δ*x*_⊥_ is the coordinate perpendicular to the GUV contour or membrane tube. The intensity profiles can be well-fitted by Gaussian distributions with a half-peak width of 0.35 ± 0.05 μm. The peak-to-peak separations for the lines 2 and 3 lead to the estimated tube diameters 2*R*_cy_ = 0.58 and 0.54 μm, respectively. Reprinted with permission from Liu et al. (2016). Copyright (2016) American Chemical Society.

Upon deflation of vesicles enclosing ATPS, three types of nanotube patterns have been observed, corresponding to three different vesicle shapes as schematically shown in Figure 10 (Liu et al., 2016). These different morphologies can be observed upon osmotic deflation of the vesicles. In the example given in Figure 10, the membrane is composed of three lipid components: dioleoylphosphatidylcholine (DOPC), dipalmitoylphosphatidylcholine (DPPC), and cholesterol (Chol), which can exhibit different phase state depending on the exact composition. Bilayer phases such as the liquid-disordered (Ld) and liquid-ordered (Lo) phases and their coexistence can be directly observed as fluid domains in GUVs (Lipowsky and Dimova, 2003) using fluorescence microscopy (Dietrich et al., 2001). In the case of GUVs enclosing ATPS (Liu et al., 2016), we employed two different membrane compositions, corresponding to a Ld membrane with lipid composition DOPC:DPPC:Chol = 64:15:21 (mole fractions) and a Lo one with lipid composition DOPC:DPPC:Chol = 13:44:43 (see Figures 11, 12), respectively. These two membranes are both in the single phase region and have different elastic property, with the bending rigidities κ_Ld_ = 0.82 × 10^−19^ J for the Ld membranes and κ_Lo_ = 3.69 × 10^−19^ J for the Lo membranes (Heinrich et al., 2010).

To obtain the observed morphologies, we prepare spherical vesicles that enclose a homogeneous solution of PEG–dextran mixture. Deflation of these vesicles is then induced by gradually exchanging the exterior solution to a hypertonic one containing fixed concentrations of the two polymer components with increasing amount of sucrose up to 15.6 mM. In this low concentration regime, the effect of sucrose on the bending rigidity and spontaneous curvature of the membranes can be neglected (Döbereiner et al., 1999; Vitkova et al., 2006; Lipowsky, 2013; Dimova, 2014). For more details of the experimental procedure, the readers are referred to the original article (Liu et al., 2016). Upon small deflation, the interior polymer solution still remains as a uniform aqueous phase with *c* < *c*_cr_ (see VM-A morphology in Figure 10), but the area needed to enwrap the (reduced) volume of the vesicle is now in excess, which result in the formation of tubes (the excess area is stored in them). Subsequent deflation steps with *c* > *c*_cr_, result in phase separation of the interior solution into two aqueous phases, a lighter PEG-rich and a heavier dextran-rich phase, both confined by the vesicles as liquid droplets. When the membrane is completely wetted by the PEG-rich droplet, the dewetted dextran-rich droplet is surrounded by the PEG-rich phase and has no contact with the membrane, which defines the VM-B morphology of the vesicles (Figure 10). The dextran-rich droplet sinks to the bottom of the vesicle because its density is always larger than the density of the coexisting PEG-rich phase (Figure 3A). Upon further deflation, both aqueous phases are in contact with the membranes, indicating a partial-wetting state of the two aqueous phases. This is defined as the VM-C morphology for the vesicles (Figure 10). The two membrane segments and the pd interface form non-zero contact angles (see Figure 9f). It is found that the complete-to-partial wetting transitions are located between different deflation steps for the Ld and Lo membranes, reflecting different wetting property of ATPS on these membranes.

Due to the different wetting properties of the aqueous phases on the membranes, different nanotube patterns formed in the VM-B and VM-C morphologies are observed by the confocal microscope (see Figure 11). For the complete wetting morphology VM-B, these nanotubes explore the interior of the whole PEG-rich droplet, and undergo strong thermally excited undulations. The length of the individual nanotubes can be estimated from stack of three-dimensional scans of the vesicles, which is on the order of 20 μm for Ld vesicles. For the partial wetting morphology VM-C, these nanotubes adhere to the pd interface between the two liquid droplets, where one can immediately see the long tube segments in a single scan. The local adhesion of the nanotubes to the liquid-liquid interface is a reflection of the complete-to-partial wetting transition.

These nanotubes can be either necklace-like consisting of a number of small spheres connected by narrow or closed membrane necks or cylindrical with a uniform diameter along the nanotube (Figure 10). Theoretical investigation of the nucleation and growth of the tubes indicated that these membrane nanotubes prefer necklace-like shape at short length but cylindrical one above a critical length, which can be understood by minimization of the membrane bending energy (Liu et al., 2016). The necklace-cylinder transformation occurs at the critical tube length of about three times of the mother vesicle radius, and the tubes can reshape themselves via a series of intermediate unduloids (Liu et al., 2016). For the partial wetting morphology VM-C, due to additional contribution from adhesion free energy of the tubes at the pd interface, the critical length for necklace-cylinder transformation depends on the material parameters and can become as low as a few micrometers. Therefore, the shape of the Ld tubes in the VM-A and VM-B morphologies are predicted to be necklace-like, but a co-existence of necklace-like and cylindrical shape is expected for Ld tubes in the VM-C morphology. In contrast, the tubes of the stiffer Lo membranes are so thick that their shapes can be directly observed from the confocal images. Necklace-like shape tubes are observed for all three morphologies of the Lo vesicles. Surprisingly, the confocal images in Figure 12 revealed the co-existence of several long cylindrical tubes and a few short necklace-like tubes at the pd interface. The length of these cylindrical tubes is above the critical length for the necklace–cylinder transformation.

### Spontaneous Curvatures of Vesicle Enclosing ATPS

**Figure 13:**
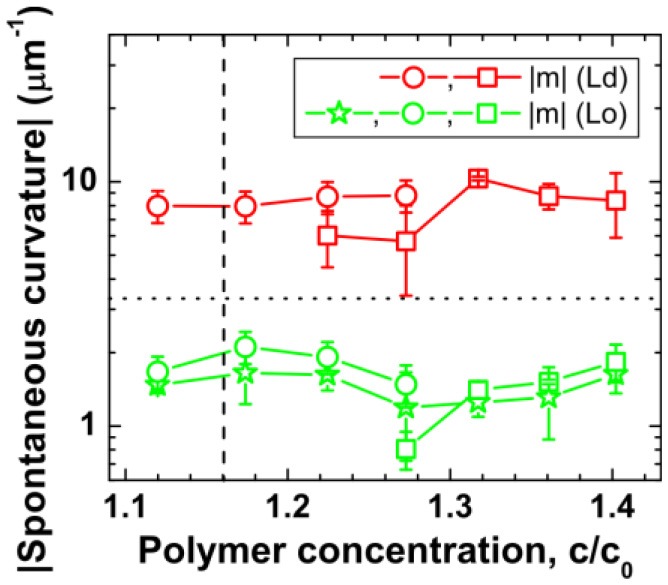
Variation of deduced spontaneous curvature of Ld (red) and Lo (green) membranes with polymer concentration modulated by osmotic deflation of the vesicles. The vertical dashed lines correspond to the critical concentration *c*_cr_. The data were obtained by direct shape analysis of the nanotubes (green stars), area partitioning analysis as given by Equation (8) (open circles), and force balance analysis described by Equation (9) (open squares). The horizontal dotted line corresponds to the optical resolution limit of 1/(300 nm). Reprinted with permission from Liu et al. (2016). Copyright (2016) American Chemical Society.

Several approaches for deducing the membrane spontaneous curvature have been developed in Li et al. (2011), Lipowsky (2013, 2014), Liu et al. (2016), Bhatia et al. (2018), and Dasgupta et al. (2018), some of which have been reviewed in section Measuring the Membrane Spontaneous Curvature of Bassereau et al. (2018). Stable membrane nanotubes were first observed for vesicles encapsulating ATPS in Li et al. (2011), and the theoretical analysis of the corresponding GUV shapes revealed the presence of a negative spontaneous curvature of about −1/(240 nm). We then developed three different and independent methods to determine this curvature based on image analysis of tubulated vesicles made of both Ld and Lo membranes (Liu et al., 2016). As shown in Figure 13, all these methods led to consistent values of the spontaneous curvatures for both Ld and Lo vesicles of three different morphologies.

The first method is based on direct measurement of the tube thickness by confocal microscopy. For the Lo membranes, the tubes have diameters well above the optical resolution, which made it possible to measure the tube size directly from the confocal scans. Short necklace-like tubes are observed for all Lo vesicles in the VM-A and VM-B morphologies, which leads to the estimate of the spontaneous curvature via *m* = −1/ <*R*_ss_>. The relative standard deviation of the radius *R*_ss_ for the quasi-spherical beads is about 20%. The direct shape analysis is also applicable for Lo tubes in the VM-C morphology where the cylindrical tubes co-exist with the necklace-like ones. For the vesicles shown in Figure 12, measurement of the average diameter <2*R*_cy_> of the cylindrical tubes leads to the spontaneous curvature *m* = −1/ <2*R*_cy_> = −1.82 μm^−1^, with an accuracy of about ±13%. While measurement of the average bead radius <*R*_ss_> of the necklace-like tubes gives the estimation of *m* = −1/ <*R*_ss_> = −1.56 μm^−1^, having an uncertainty of about ±19%. Interestingly, nearly identical *m*-values for the cylindrical and necklace-like tubes formed from the same vesicle are obtained, indicating the uniformity of the membrane spontaneous curvature. Thus, the spontaneous curvatures of Lo membranes are determined by the direct shape analysis for all vesicle morphologies, and the results are shown in Figure 13 as green stars. This method is, however, not applicable for Ld tubes, because they are so thin that their shape is not resolvable by the confocal microscope.

The second method is based on the membrane area partitioning between nanotubes and the mother vesicle. The shapes of the nanotubes for Ld vesicles cannot be resolved by confocal microscope because the tube diameter is below the optical resolution. But we can calculate the spontaneous curvature via two measurable geometric quantities: the area *A* and length *L* of all tubes. It is based on the fact that the excess area generated by deflation is stored as nanotubes. Upon deflation, the vesicle apparent area *A*_app_ is less than the initial vesicle area *A*_0_, both areas can be obtained from the vesicle shape and their difference (*A*_0_ –*A*_app_) is the missing area stored as tubes. While the length *L* can be measured from 3D scans of the vesicle by confocal microscope. Then the spontaneous curvature of the membrane can be estimated via (Liu et al., 2016):

(8) $$\begin{array}{r} m=-\left(2-\Lambda \right)\pi L/A \end{array}$$

Here Λ is the fraction of the total tube length in cylindrical shape, and the rest part is necklace-like.

For short necklace-like tubes observed for all Lo vesicles in the VM-A and VM-B morphologies, Λ = 0 is obtained. For Lo tubes in the VM-C morphology with a co-existence of the cylindrical and necklace-like tubes, non-zero Λ-value is observed. However, for the Ld tubes with thickness below the optical resolution, the fraction Λ cannot be estimated from the confocal images. These flexible Ld tubes in the VM-A and VM-B morphologies are predicted to be necklace-like, which leads to Λ = 0. For Ld tubes in the VM-C morphology, a co-existence of the cylindrical and necklace-like tubes is expected, but one cannot estimate the fraction Λ. In this case, we have to take all possible Λ-values into account. The spontaneous curvatures for the Ld membranes are then estimated using Equation (8) with Λ = 0 for VM-A and VM-B morphologies and 0 ≤ Λ ≤ 1 for VM-C morphology. The *m*-values obtained by area partitioning analysis for both Lo and Ld membranes are shown in Figure 13 as green and red circles, respectively. The accuracy of this method is ±15%, resulting mainly from the uncertainty of the measured tube length *L*. It should be noted that when the tubes are too crowded at the pd interface for the highest polymer concentrations of VM-C morphology, it becomes rather difficult to estimate the total tube length and then this method is not applicable.

For the VM-C morphologies, where two membrane segments and the pd interface form non-zero contact angles due to partial wetting of the aqueous phases, the membrane spontaneous curvature can be estimated via a third method based on force balance of the tensions at the three-phase contact line. Since the tubes are always protruded into the PEG-rich phase and adhere to the liquid-liquid interface for VM-C morphology, one can estimate the spontaneous curvature via (Lipowsky, 2013, 2014; Liu et al., 2016):

(9) $$\begin{array}{r} m=-{\left(\frac{\Sigma_{\text{pd}}}{2\kappa }\frac{\sin\theta_{d}}{\sin\theta_{e}}\right)}^{1/2} \end{array}$$

Here κ is the bending rigidity of membrane. One can calculate the *m*-values for both Lo and Ld membranes, with the separately measured interfacial tension Σ_pd_, the effective contact angles θ_d_ and θ_e_, and the bending rigidities κ_Lo_ and κ_Ld_ (Heinrich et al., 2010). The obtained results are shown in Figure 13 as green and red squares, respectively. It is obvious that all three modes of image analysis led to consistent values for the spontaneous curvatures of these membranes.

It should be noted that the spontaneous curvatures of these two membranes were found to be almost constant, with *m*_Ld_ ≅ – 8 μm^−1^ and *m*_Lo_ ≅ – 1.7 μm^−1^ over the range of studied polymer concentrations. Their spontaneous curvature ratio of *m*_Ld_/*m*_Lo_ ≅ 4.7 is nearly identical to their bending rigidity ratio of κ_Lo_/κ_Ld_ ≅ 4.5. The observed inverse proportionality between the spontaneous curvature and the bending rigidity is in accord with the generation of these curvatures by adsorption, as shown in next section.

### Molecular Mechanism of Curvature Generation in Vesicles Enclosing ATPS

Because the formation of nanotubes in the GUVs was observed only in the presence of polymers, the spontaneous curvature of the vesicle membranes should be generated by the interactions between membrane and the encapsulated polymers. Depending on the effectively attractive or repulsive force with the membranes, polymer molecules can form either adsorption or depletion layers on the membrane, and result in bulging of the lipid bilayer toward the solution with higher concentration of polymer adsorption or lower concentration of polymer depletion.

In all three vesicle morphologies shown in Figure 10, the concentration of PEG in the interior solution is always larger than that in the exterior solution. However, the concentration of dextran in the interior solution is larger for VM-A morphology but smaller for VM-B and VM-C morphologies than the exterior dextran concentration. At the same time, all deflation steps led to the formation of tubes protruding into the interior of vesicles with a negative spontaneous curvature. Therefore, the observation is consistent with the theoretical prediction (Breidenich et al., 2005), only if the spontaneous curvature is generated by adsorption of PEG onto the membrane. This conclusion was supported by control experiments with both Ld and Lo vesicles enclosing pure PEG solution without dextran. Deflation of these vesicles led to nanotubes protruding into the interior of the vesicles with higher PEG concentration.

**Figure 14:**
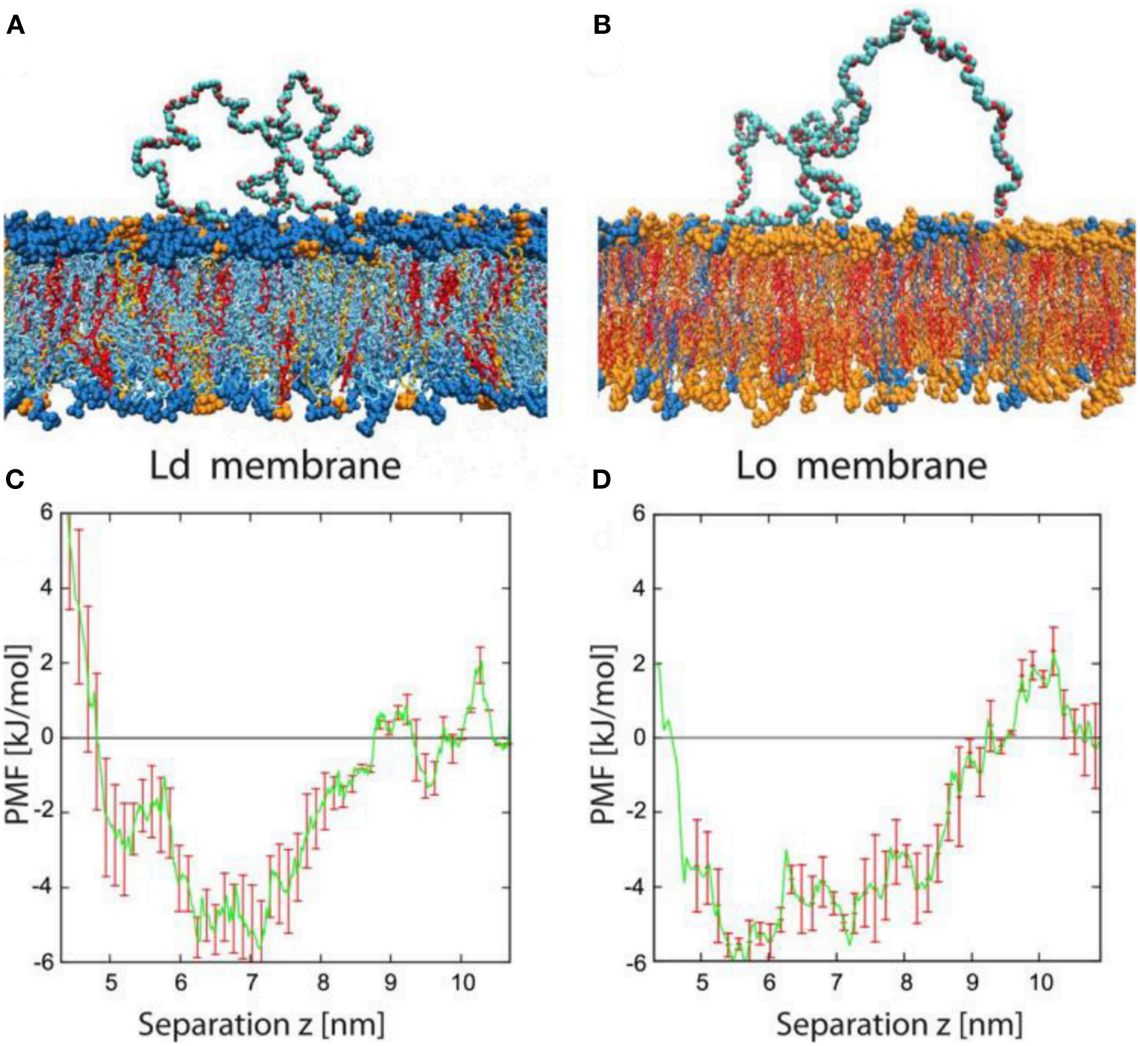
Typical conformation and potential of mean force for adsorbed PEG molecules. **(A,B)** Simulation snapshots of PEG molecule adsorbed onto Ld and Lo bilayer. The color code for the lipids is blue for DOPC, orange for DPPC, and red for cholesterol. The PEG molecules consist of 180 monomers corresponding to the average molecular weight used in the experiments. Each lipid membrane is immersed in about 27,000 water molecules (not shown). **(C,D)** Potential of mean force (PMF) for Ld and Lo membranes as a function of the separation *z* between the polymer's center-of-mass and the bilayer midplane. The potential wells are relatively broad, with a width of about 4 nm, because the polymer end groups can adsorb even for relatively large *z*-values. The binding free energy of a single PEG chain is about 4 kJ/mol or 1.6 k_B_T for both types of membranes. Reprinted with permission from Liu et al. (2016). Copyright (2016) American Chemical Society.

To further elucidate conformations of the PEG chains adsorbed on the membranes and the role of PEG-membrane interactions on the curvature generation of the membranes, we performed atomistic molecular dynamics simulations on the same hybrid lipid-polymer systems as in the experiments (Liu et al., 2016). Typical conformations of the PEG molecules adsorbed onto the Ld and Lo membranes are shown in Figures 14A,B. It indicated that PEG chains are only weakly bound to the membranes, with long loops dangled between some short adsorption segments. It is often observed that the PEG chain binds to the membrane by hydrogen bonds formed between the two terminal OH groups and the head groups of the lipid. Less frequently, a few contacts form between the PEG backbones and the membranes. The affinity of the PEG molecules to the membranes is further quantified by the potentials of mean force, as shown in Figures 14C,D. It indicated that the studied PEG chains have the same binding affinity to the Ld and Lo membranes, with a relatively small binding energy of about 4 kJ/mol or 1.6 k_B_T per PEG molecule. It is consistent with the experimental results where the spontaneous curvature ratio *m*_Ld_/*m*_Lo_ is equal to the bending rigidity ratio κ_Lo_/κ_Ld_.

## Conclusions

In summary, we discussed the model system of GUVs encapsulating ATPS emphasizing aspects of both polymer physics and membrane biophysics, highlighting recent results from our groups.

We illustrated how the phase diagram for ATPS of dextran and PEG can be constructed by cloud titration and presented methods based on density and GPC measurements of the coexisting phases. The ultralow interfacial tension between the coexisting phases was studied over a broad polymer concentration range above the critical point. It was found that the scaling exponent of the interfacial tension with the reduced polymer concentration gives a value of 1.67 in vicinity of the critical point, which disagrees with the expected value 1.26 for the Ising model. The latter discrepancy arises from the molar mass fractionation of dextran during phase separation.

When encapsulating these ATPS into giant vesicles, the membranes may be completely or partially wetted by the two aqueous phases, depending on the lipid and polymer composition. A complete-to-partial wetting transition of ATPS is observed via osmotic deflation of the vesicle volume. The associated volume reduction generates excess area of the membrane, which folds into many membrane nanotubes protruding into the interior vesicle compartment revealing a substantial asymmetry and negative spontaneous curvature of the membranes. Quantitative estimates of the spontaneous curvature have been obtained in Liu et al. (2016) by three different and independent methods of image analysis. The spontaneous curvature is generated by the weak PEG adsorption onto the lipid membranes, with a binding affinity of about 1.6 k_B_T per PEG molecule for either liquid-ordered or liquid-disordered membranes, based on molecular dynamics simulation.

Membrane nanotubes are also observed in the living cells, for example in the Golgi apparatus and the smooth endoplasmic reticulum. However, the underlying mechanism for the tube formation in cells remains to be elucidated. The cellular membranes are often exposed to asymmetric aqueous environments with a large amount of proteins, which plays central role in the tubulation process. The model system of GUV encapsulating ATPS provides a controllable platform for understanding the remodeling of membranes in the living cells. It would be interesting to include proteins in the GUV/ATPS system to mimic the cellular behavior more closely.

## Author Contributions

All authors listed have made a substantial, direct and intellectual contribution to the work, and approved it for publication.

### Conflict of Interest Statement

The authors declare that the research was conducted in the absence of any commercial or financial relationships that could be construed as a potential conflict of interest. The reviewer PS declared a past co-authorship with one of the authors RD to the handling editor.

## References

[B1] Albertsson P. Å. (1986). Partition of Cell Particles and Macromolecules: Separation and Purification of Biomolecules, Cell Organelles, Membranes, and Cells in Aqueous Polymer Two-Phase Systems and Their Use in Biochemical Analysis and Biotechnology. New York, NY: Wiley.

[B2] Andes-Koback M. Keating C. D. (2011). Complete budding and asymmetric division of primitive model cells to produce daughter vesicles with different interior and membrane compositions. J. Am. Chem. Soc. 133, 9545–9555. 10.1021/ja202406v 21591721 PMC3115689

[B3] Anisimov M. A. Agayan V. A. Gorodetskii E. E. (2000). Scaling and crossover to tricriticality in polymer solutions. JETP Lett. 72, 578–582. 10.1134/1.1348485

[B4] Anisimov M. A. Kostko A. F. Sengers J. V. (2002). Competition of mesoscales and crossover to tricriticality in polymer solutions. Phys. Rev. E 65, 051805. 10.1103/PhysRevE.65.051805 12059586

[B5] Atefi E. Mann J. A. Tavana H. (2014). Ultralow interfacial tensions of aqueous two-phase systems measured using drop shape. Langmuir 30, 9691–9699. 10.1021/la500930x 25068649

[B6] Bassereau P. Jin R. Baumgart T. Deserno M. Dimova R. Frolov V. A. (2018). The 2018 biomembrane curvature and remodeling roadmap. J. Phys. D Appl. Phys. 51, 343001. 10.1088/1361-6463/aacb98 30655651 PMC6333427

[B7] Bhatia T. Agudo-Canalejo J. Dimova R. Lipowsky R. (2018). Membrane nanotubes increase the robustness of giant vesicles. ACS Nano 12, 4478–4485. 10.1021/acsnano.8b00640 29659246

[B8] Breidenich M. Netz R. Lipowsky R. (2005). The influence of non-anchored polymers on the curvature of vesicles. Mol. Phys. 103, 3169–3183. 10.1080/00268970500270484

[B9] Connemann M. Gaube J. Leffrang U. Muller S. Pfennig A. (1991). Phase equilibria in the system poly(ethylene glycol) + dextran + water. J. Chem. Eng. Data 36, 446–448. 10.1021/je00004a029

[B10] Dasgupta R. Miettinen M. Fricke N. Lipowsky R. Dimova R. (2018). The glycolipid GM1 reshapes asymmetric biomembranes and giant vesicles by curvature generation. Proc. Natl. Acad. Sci. U.S.A. 115, 5756–5761. 10.1073/pnas.1722320115 29760097 PMC5984512

[B11] Dietrich C. Bagatolli L. A. Volovyk Z. N. Thompson N. L. Levi M. Jacobson K.. (2001). Lipid rafts reconstituted in model membranes. Biophys. J. 80, 1417–1428. 10.1016/S0006-3495(01)76114-0 11222302 PMC1301333

[B12] Dimova R. (2012). Giant vesicles: a biomimetic tool for membrane characterization, in Advances in Planar Lipid Bilayers and Liposomes, ed Iglic A. (Amsterdam: Academic Press), 1–50.

[B13] Dimova R. (2014). Recent developments in the field of bending rigidity measurements on membranes. Adv. Coll. Interf. Sci. 208, 225–234. 10.1016/j.cis.2014.03.003 24666592

[B14] Dimova R. (2019). Giant vesicles and their use in assays for assessing membrane phase state, curvature, mechanics and electrical properties. Annu. Rev. Biophys. 48:1 10.1146/annurev-biophys-052118-115342 30811220

[B15] Dimova R. Aranda S. Bezlyepkina N. Nikolov V. Riske K. A. Lipowsky R. (2006). A practical guide to giant vesicles. Probing the membrane nanoregime via optical microscopy. J. Phys. Condens. Matter 18, S1151–S1176. 10.1088/0953-8984/18/28/S04 21690835

[B16] Dimova R. Lipowsky R. (2012). Lipid membranes in contact with aqueous phases of polymer solutions. Soft Matter 8, 6409–6415. 10.1039/c2sm25261a

[B17] Dimova R. Lipowsky R. (2017). Giant vesicles exposed to aqueous two-phase systems: membrane wetting, budding processes, and spontaneous tubulation. Adv. Mater. Interfaces 4, 1600451 10.1002/admi.201600451

[B18] Ding P. Wolf B. Frith W. J. Clark A. H. Norton I. T. Pacek A. W. (2002). Interfacial tension in phase-separated gelation/dextran aqueous mixtures. J. Colloid Interface Sci. 253, 367–376. 10.1006/jcis.2002.8572 16290867

[B19] Dobashi T. Nakata M. Kaneko M. (1980). Coexistence curve of polystyrene in methylcyclohexane. 2. Comparison of coexistence curve observed and calculated from classical free-energy. J. Chem. Phys. 72, 6692–6697. 10.1063/1.439128

[B20] Döbereiner H. G. Selchow O. Lipowsky R. (1999). Spontaneous curvature of fluid vesicles induced by trans-bilayer sugar asymmetry. Eur. Biophys. J. 28, 174–178. 10.1007/s002490050197

[B21] Edelman M. W. Tromp R. H. van der Linden E. (2003a). Phase-separation-induced fractionation in molar mass in aqueous mixtures of gelatin and dextran. Phys. Rev. E 67, 021404. 10.1103/PhysRevE.67.021404 12636676

[B22] Edelman M. W. van der Linden E. Tromp R. H. (2003b). Phase separation of aqueous mixtures of poly(ethylene oxide) and dextran. Macromolecules 36, 7783–7790. 10.1021/ma0341622

[B23] Flory P. J. (1941). Thermodynamics of high polymer solutions. J. Chem. Phys. 9, 660–661. 10.1063/1.1750971

[B24] Flory P. J. (1953). Principles of Polymer Chemistry. Ithaca: Cornell University Press.

[B25] Hatti-Kaul R. (2000). Methods in Biotechnology, Vol. 11, Aqueous Two-Phase Systems: Methods and Protocols. Totowa: Humana Press.

[B26] Heinrich M. Tian A. Esposito C. Baumgart T. (2010). Dynamic sorting of lipids and proteins in membrane tubes with a moving phase boundary. Proc. Natl. Acad. Sci. U.S.A. 107, 7208–7213. 10.1073/pnas.0913997107 20368457 PMC2867702

[B27] Heinrich M. Wolf B. A. (1992). Interfacial tension between solutions of polystyrenes: establishment of a useful master curve. Polymer 33, 1926–1931. 10.1016/0032-3861(92)90494-H

[B28] Helfrich M. R. Mangeney-Slavin L. K. Long M. S. Djoko K. Y. Keating C. D. (2002). Aqueous phase separation in giant vesicles. J. Am. Chem. Soc. 124, 13374–13375. 10.1021/ja028157+ 12418876

[B29] Huggins M. L. (1941). Solutions of long chain compounds. J. Chem. Phys. 9, 440 10.1063/1.1750930

[B30] Kang C. H. Sandler S. I. (1988). Effects of polydispersivity on the phase behavior of the aqueous two-phase polymer systems. Macromolecules 21, 3088–3095. 10.1021/ma00188a029

[B31] Keating C. D. (2012). Aqueous phase separation as a possible route to compartmentalization of biological molecules. Acc. Chem. Res. 45, 2114–2124. 10.1021/ar200294y 22330132 PMC3525015

[B32] Koningsveld R. Stockmayer W. H. Nies E. (2001). Polymer Phase Diagrams. New York, NY: Oxford University Press.

[B33] Kusumaatmaja H. Li Y. Dimova R. Lipowsky R. (2009). Intrinsic contact angle of aqueous phases at membranes and vesicles. Phys. Rev. Lett. 103, 238103. 10.1103/PhysRevLett.103.238103 20366179

[B34] Li Y. Kusumaatmaja H. Lipowsky R. Dimova R. (2012). Wetting-induced budding of vesicles in contact with several aqueous phases. J. Phys. Chem. B 116, 1819–1823. 10.1021/jp211850t 22242924 PMC3280617

[B35] Li Y. Lipowsky R. Dimova R. (2008). Transition from complete to partial wetting within membrane compartments. J. Am. Chem. Soc. 130, 12252–12253. 10.1021/ja8048496 18712871

[B36] Li Y. Lipowsky R. Dimova R. (2011). Membrane nanotubes induced by aqueous phase separation and stabilized by spontaneous curvature. Proc. Natl. Acad. Sci. U.S.A. 108, 4731–4736. 10.1073/pnas.1015892108 21383120 PMC3064332

[B37] Lipowsky R. (2013). Spontaneous tubulation of membranes and vesicles reveals membrane tension generated by spontaneous curvature. Faraday Discuss. 161, 305–331. 10.1039/c2fd20105d 23805747

[B38] Lipowsky R. (2014). Remodeling of membrane compartments: some consequences of membrane fluidity. Biol. Chem. 395, 253–274. 10.1515/hsz-2013-0244 24491948

[B39] Lipowsky R. (2018). Response of membranes and vesicles to capillary forces arising from aqueous two-phase systems and water-in-water droplets. J. Phys. Chem. B 122, 3572–3586. 10.1021/acs.jpcb.7b10783 29465241

[B40] Lipowsky R. Dimova R. (2003). Domains in membranes and vesicles. J. Phys. Condens. Matter 15, S31–S45. 10.1088/0953-8984/15/1/304

[B41] Liu Y. Agudo-Canalejo J. Grafmüller A. Dimova R. Lipowsky R. (2016). Patterns of flexible nanotubes formed by liquid-ordered and liquid-disordered membranes. ACS Nano 10, 463–474. 10.1021/acsnano.5b05377 26588094

[B42] Liu Y. Lipowsky R. Dimova R. (2012). Concentration dependence of the interfacial tension for aqueous two-phase polymer solutions of dextran and polyethylene glycol. Langmuir 28, 3831–3839. 10.1021/la204757z 22292882

[B43] Long M. S. Cans A. S. Keating C. D. (2008). Budding and asymmetric protein microcompartmentation in giant vesicles containing two aqueous phases. J. Am. Chem. Soc. 130, 756–762. 10.1021/ja077439c 18092782

[B44] Long M. S. Jones C. D. Helfrich M. R. Mangeney-Slavin L. K. Keating C. D. (2005). Dynamic microcompartmentation in synthetic cells. Proc. Natl. Acad. Sci. U.S.A. 102, 5920–5925. 10.1073/pnas.0409333102 15788532 PMC1087917

[B45] Melnichenko Y. B. Anisimov M. A. Povodyrev A. A. Wignall G. D. Sengers J. V. van Hook W. A. (1997). Sharp crossover of the susceptibility in polymer solutions near the critical demixing point. Phys. Rev. Lett. 79, 5266–5269. 10.1103/PhysRevLett.79.5266

[B46] Merchuk J. C. Andrews B. A. Asenjo J. A. (1998). Aqueous two-phase systems for protein separation studies on phase inversion. J. Chromatogr. B 711, 285–293. 10.1016/S0378-4347(97)00594-X 9699997

[B47] Mishima K. Matsuyama K. Ezawa M. Taruta Y. Takarabe S. Nagatani M. (1998). Interfacial tension of aqueous two-phase systems containing poly(ethylene glycol) and dipotassium hydrogenphosphate. J. Chromatogr. B 711, 313–318. 10.1016/S0378-4347(97)00660-9 9700001

[B48] Ryden J. Albertsson P. A. (1971). Interfacial tension of dextran-polyethylene glycol-water two-phase systems. J. Colloid Interface Sci. 37, 219–222. 10.1016/0021-9797(71)90283-9

[B49] Sanchez I. C. (1989). Critical amplitude scaling laws for polymer solutions. J. Phys. Chem. 93, 6983–6991. 10.1021/j100356a021

[B50] Shinozaki K. Vantan T. Saito Y. Nose T. (1982). Interfacial tension of demixed polymer solutions near the critical temperature: polystyrene + methylcyclohexane. Polymer 23, 728–734. 10.1016/0032-3861(82)90059-3

[B51] Tromp R. H. (2016). Water-water interphases, in Soft Matter at Aqueous Interfaces. Lecture Notes in Physics, eds Lang P. Liu Y. (Cham: Springer), 159–186.

[B52] van Heukelum A. Barkema G. T. Edelman M. W. van der Linden E. de Hoog E. H. A. Tromp R. H. (2003). Fractionation in a phase-separated polydisperse polymer mixtures. Macromolecules 36, 6662–6667. 10.1021/ma025736q

[B53] Vis M. Peters V. F. D. Blokhuis E. M. Lekkerkerker H. N. W. Erne B. H. Tromp R. H. (2015). Effects of electric charge on the interfacial tension between coexisting aqueous mixtures of polyelectrolyte and neutral polymer. Macromolecules 48, 7335–7345. 10.1021/acs.macromol.5b01675

[B54] Vitkova V. Genova J. Mitov M. D. Bivas I. (2006). Sugars in the aqueous phase change the mechanical properties of lipid mono- and bilayers. Mol. Cryst. Liq. Cryst. 449, 95–106. 10.1080/15421400600582515

[B55] Walde P. Cosentino K. Engel H. Stano P. (2010). Giant vesicles: preparations and applications. ChemBioChem 11, 848–865. 10.1002/cbic.201000010 20336703

[B56] Walter H. Brooks D. E. Fisher D. (1985). Partitioning in Aqueous Two-phase Systems: Theory, Methods, Uses, and Applications to Biotechnology. Orlando: Academic Press.

[B57] Widom B. (1993). Phase separation in polymer solutions. Phys. A 194, 532–541. 10.1016/0378-4371(93)90383-F

[B58] Zaslavski B. Y. (1995). Aqueous Two-Phase Partitioning: Physical Chemistry and Bioanalytical Applications. New York, NY: Marcel Dekker.

[B59] Zhao Z. Li Q. Ji X. Dimova R. Lipowsky R. Liu Y. (2016a). Molar mass fractionation in aqueous two-phase polymer solutions of dextran and poly(ethylene glycol). J. Chromatogr. A 1452, 107–115. 10.1016/j.chroma.2016.04.075 27155914

[B60] Zhao Z. Li Q. Xue Y. Ji X. Bo S. Liu Y. (2016b). Composition and molecular weight determination of aqueous two-phase system by quantitative size exclusion chromatography, Chem. J. Chin. Univ. 37, 167–173. 10.7503/cjcu20150567

[B61] Zhao Z. Roy D. Steinkühler J. Robinson T. Knorr R. Lipowsky R. (2018). Super resolution imaging of highly curved membrane structures in giant unilamellar vesicles encapsulating polymer solutions. Biophys. J. 114, 100a−101a. 10.1016/j.bpj.2017.11.591

